# Ancient mitochondrial diversity reveals population homogeneity in Neolithic Greece and identifies population dynamics along the Danubian expansion axis

**DOI:** 10.1038/s41598-022-16745-8

**Published:** 2022-08-05

**Authors:** Nuno M. Silva, Susanne Kreutzer, Angelos Souleles, Sevasti Triantaphyllou, Kostas Kotsakis, Dushka Urem-Kotsou, Paul Halstead, Nikos Efstratiou, Stavros Kotsos, Georgia Karamitrou-Mentessidi, Fotini Adaktylou, Areti Chondroyianni-Metoki, Maria Pappa, Christina Ziota, Adamantios Sampson, Anastasia Papathanasiou, Karen Vitelli, Tracey Cullen, Nina Kyparissi-Apostolika, Andrea Zeeb Lanz, Joris Peters, Jérémy Rio, Daniel Wegmann, Joachim Burger, Mathias Currat, Christina Papageorgopoulou

**Affiliations:** 1 Department of Genetics & Evolution, University of Geneva, Geneva, Switzerland; 2 Palaeogenetics Group, Institute of Organismic and Molecular Evolution (iomE), Johannes Gutenberg University of Mainz, 55099 Mainz, Germany; 3 Laboratory of Physical Anthropology, Department of History & Ethnology, Democritus University of Thrace, 69100 Komotini, Greece; 4 Faculty of Philosophy, School of History and Archaeology, Aristotle University of Thessaloniki, 54124 Thessaloniki, Greece; 5 Department of History & Ethnology, Democritus University of Thrace, 69100 Komotini, Greece; 6 Emeritus, Department of Archaeology, University of Sheffield, Sheffield, S1 3NJ UK; 7 Ephorate of Antiquities of Thessaloniki City, Hellenic Ministry of Culture and Sports, 54003 Thessaloniki, Greece; 8 Ephor Emerita of Antiquities, Hellenic Ministry of Culture & Sports, 10682 Athens, Greece; 9 Ephorate of Antiquities of Chalcidice and Mount Athos, Hellenic Ministry of Culture and Sports, 63100 Poligiros Chalcidice, Greece; 10 Ephorate of Antiquities of Kozani, Hellenic Ministry of Culture and Sports, 50131 Kozani, Greece; 11 Ephorate of Antiquities of Thessaloniki Region, Hellenic Ministry of Culture and Sports, 54646 Thessaloniki, Greece; 12 Ephorate of Antiquities of Florina, Hellenic Ministry of Culture and Sports, 53100 Florina, Greece; 13 Department of Mediterranean Studies, University of Aegean, 85132 Rhodes, Greece; 14 Ephorate of Paleoanthropology and Speleology, Hellenic Ministry of Culture and Sports, 11636 Athens, Greece; 15 Prof. Emerita, Department of Anthropology, Franchthi Cave Project, Indiana University Bloomington, Bloomington, USA; 16 American School of Classical Studies at Athens, Princeton, NJ USA; 17 Ephor Emerita of the Ephorate of Paleoanthropology and Speleology, Hellenic Ministry of Culture and Sports, 11636 Athens, Greece; 18 General Direction for Cultural Heritage of Rhineland-Palatinate, Speyer, Germany; 19 Institute of Palaeoanatomy, Domestication Research and the History of Veterinary Medicine, LMU Munich, Munich, Germany; 20 SNSB, State Collection of Palaeoanatomy Munich, Munich, Germany; 21 Department of Biology, University of Fribourg, 1700 Fribourg, Switzerland; 22 Swiss Institute of Bioinformatics, 1700 Fribourg, Switzerland; 23 Institute of Genetics and Genomics in Geneva (IGE3), University of Geneva, Geneva, Switzerland; 24 Present Address: Functional Genomics Center Zurich/GEML, Department of Biology, ETH Zurich, Zurich, Switzerland

**Keywords:** Computational biology and bioinformatics, Evolution, Genetics

## Abstract

The aim of the study is to investigate mitochondrial diversity in Neolithic Greece and its relation to hunter-gatherers and farmers who populated the Danubian Neolithic expansion axis. We sequenced 42 mitochondrial palaeogenomes from Greece and analysed them together with European set of 328 mtDNA sequences dating from the Early to the Final Neolithic and 319 modern sequences. To test for population continuity through time in Greece, we use an original structured population continuity test that simulates DNA from different periods by explicitly considering the spatial and temporal dynamics of populations. We explore specific scenarios of the mode and tempo of the European Neolithic expansion along the Danubian axis applying spatially explicit simulations coupled with Approximate Bayesian Computation. We observe a striking genetic homogeneity for the maternal line throughout the Neolithic in Greece whereas population continuity is rejected between the Neolithic and present-day Greeks. Along the Danubian expansion axis, our best-fitting scenario supports a substantial decrease in mobility and an increasing local hunter-gatherer contribution to the gene-pool of farmers following the initial rapid Neolithic expansion. Οur original simulation approach models key demographic parameters rather than inferring them from fragmentary data leading to a better understanding of this important process in European prehistory.

## Introduction

In the last decade, ancient DNA (aDNA) studies have provided first insights into the genetic diversity and population structure of hunter-gatherers and early farmers in Europe and southwestern Asia, leading to a better understanding of the process of Neolithisation^1–8^. These palaeogenomic data imply immigration of Early Neolithic farmers from southwestern Asia to Europe^3,9,10^ following two major routes: a maritime route along the Mediterranean coastline and a mainland route along the Danube, connecting central Anatolia, Greece, the Balkans and central Europe^3,11,12^. Although this general pattern of the spread of agriculture, along with its significant demographic and socioeconomic implications, is well established today, little is known regarding regional heterogeneities.

Particularly, the region of present-day Greece at the crossroads of southwestern Asia, the Balkans, and the eastern Mediterranean, played an important role in the Neolithisation of Europe. Farming reached present-day Greece around c. 6,700 BCΕ. The earliest Neolithic sites are found on the island of Crete^13,14^, in the Peloponnese (Franchthi cave Initial Neolithic strata, 7028–6648 cal BCE^15^; Alepotrypa Cave, 6220–6030 cal BCE^16^) central Greece (Sarakenos Cave Initial Νeolithic, 6976–6685 cal BCE^17,18^) and Macedonia (Mavropigi-Fillotsairi and Paliambela-Kolindrou date to 6700–6600 BCE^19–21^. Available dates for Thessaly, from the sites of Argissa, Gendiki and Sesklo are slightly later at 6500–6400 BCΕ^22,23^. The newest archaeological findings and radiocarbon dates from northern Greece suggest that initial Neolithisation possibly occurred almost simultaneously on both sides of the Aegean^20^. From there, the Neolithic dispersal reached the northern Balkans and central Europe following three major routes: (1) the Struma Basin^24^, (2) Thrace^25^ and (3) the Black Sea^26^.

Overall, the Neolithic in Greece spans a period of nearly 4,000 years and comprises four main chronological phases, i.e., Early (6700/6500–6000/5600 BCE), Middle (6000/5600–5400/5300 BCE), Late (5400/5300–4700/4300 BCE), and Final Neolithic (4700/4300–3300/3100 BCE)^19,27–29^. The preceding Mesolithic period starts with the onset of the Holocene but is represented by only few human burials with dates spanning from 8600 to 6500 BCΕ. These findings derive from caves *i.e.*, Franchthi in the Peloponnese, Theopetra in Thessaly, Cyclops Cave on Youra island, Sarakenos cave in Boeοtia^15,18,30–35,37,38^ and open-air sites i.e., Maroulas on the island of Kythnos, 8800–8700 BC^35^. Lithic technology from the Final Paleolithic site of Ouriakos on Lemnos, an island in the northeastern Aegean (10,500 cal BCΕ^36^) and Maroulas, a Mesolithic settlement on Kythnos in the southwestern Aegean (8500–6500 BCE^37,38^) indicate cultural contacts with hunter-gatherers of southwestern Anatolian cave sites of Öküzini, Direkli, and Girmeler respectively^39,40^. This supports the hypothesis of a coastal movement of hunter-gatherers across southeastern Mediterranean^18,41–43^ in parallel to the rise of sedentary communities in central Anatolia (Așikli, Boncuklu, Pinarbaṣi^44,45^).

In Greece, farming is considered to have spread rapidly, with most settlements being established between 6600 and 6400 calBC. This is inferred by the simultaneous appearance of the “Neolithic package”, *i.e.*, the set of domesticated animals (sheep, goat, pig and cattle) and crops (wheat, barley, pulses), pottery, ground-stone artefacts, schematic figurines and the increasing number of Early Neolithic sites, particularly when compared to the previous sparsely populated Mesolithic period. Similarities in material culture (clay stamps, schematic figurines, ear-plugs, hooks) with the Near East have supported the idea of a migration of Near Eastern farmers^15^, but these parallels point variously to the Levant or central Anatolia and in some cases remain contextually isolated^15^. For example, rectangular or clustered houses, painted floors and walls, which are characteristic features of central and western Anatolia, are strikingly absent in Early Neolithic Greece^15,46^. Moreover, lithic industry from Early Neolithic Knossos shows common features to the Mesolithic of the Aegean islands, and pre-Neolithic flake industries from Cyprus^47^.

The ambiguous references to different pre-Neolithic and Neolithic landscapes underline the complexity of the process of Neolithisation in the Aegean and the difficulty of identifying a single source region^15^. Having said that, it is plausible that two or more waves of farmers, one following an island-coastal dispersal route originating in the Levant and another mainland route originating in central Anatolia, met in the Aegean. The selective and at the same time heterogeneous nature of Early Neolithic material culture in the Aegean has variously been interpreted as a deliberate loss of cultural identity^48^, a loss of cultural diversity from the core to the periphery^49^, or as a consequence of migrations in the Aegean, predating the Neolithic expansion, resulting in considerable variability and hybridity of cultural forms^46^.

Throughout the Neolithic, material culture changed as seen in ceramic traditions^50^, burial customs^51^ and lithic technology^52^. From the Middle Neolithic (6000/5600–5300 BCE) onward, a remarkable increase is observed in the number of settlements, even in less favourable environments^28,53^. The formation of more and larger communities and the use of secondary products of animals including traction and milk, promoted more complex social structures and larger economic networks. Communities were not isolated and networks of communication are already observed since the beginning of the Neolithic^46^. During the Early Neolithic, however, the networking inferred from the distribution of ceramics was indicative of local exchange between neighbouring communities, whereas by the Late Neolithic stable networks were established over a radius of at least 200 km^54–57^. Intensification of this trend during the Final Neolithic laid one of the key foundations for the development of the next major cultural transformation, namely that of the Bronze Age (BA).

From a population genetics perspective, this important period in European prehistory has only been marginally explored. Although hundreds of ancient genomes are available from southeastern Europe for a period spanning 12,000 to 500 BC^7,8,58–60^, only a single Early (Revenia^3^) and seven Late/Final Neolithic genomes from northern (Paliambela, Kleitos^3^) and southern Greece (Alepotrypa Diros^61^, Franchthi Caves^8^) are currently available. The genome from Early Neolithic Revenia in northern Greece shows strong similarities to human genomes from contemporaneous sites in northwestern Anatolia (*i.e.*, Barcιn^3^) and to early farmers in central and western Europe. The Final Neolithic genomes show an additional signal of gene-flow with a population that has genomic affinities with hunter-gatherers from the Caucasus^3,8^. Moreover, the mitochondrial haplogroups of the only two Mesolithic individuals analysed from the Aegean so far, belong to lineages reported in central Anatolian and Aegean Neolithic populations, but not in central and western European hunter-gatherers^3^.

As the area of present-day Greece constitutes the first stepping-stone in the spread of agriculture towards Europe, human genetic diversity and genetic differentiation between individuals from different Neolithic periods can be expected to be particularly informative regarding the dynamics of the Neolithisation process. Here, we sampled 70 individuals from three Mesolithic and 12 Neolithic sites across Greece covering the period from 7050 to 3300 BCE (Supplementary Information). We were able to acquire mitochondrial genomes from 42 Neolithic individuals and document diachronic genetic diversity in relation to other European Mesolithic, Neolithic, and modern populations. Together with 18 newly reported and 310 previously published mitochondrial genomes from the Balkans and central Europe, we evaluated our dataset in order to enhance insight into population dynamics along the Danubian route using a spatially explicit computational simulation approach and Approximate Bayesian Computation (ABC). In particular, we wanted to clarify whether the higher amount of hunter-gatherer ancestry observed in the later Neolithic stage^62,63^ resulted from admixture at a constant rate or whether this rate increased over time, meaning that more and more people with hunter-gatherer ancestry became integrated into farming communities. In addition, we also wanted to investigate whether the fast migration of early farmers from the Aegean area towards central Europe^3,64^ was accompanied by substantial admixture with hunter-gatherers in the early stage.

## Material and methods

### Sample preparation and enrichment of the mitochondrial genomes

Ancient DNA (aDNA) analysis of prehistoric specimens was performed in the dedicated cleanroom facilities of the Palaeogenetics group at the University of Mainz, Germany. In total we sampled nine individuals from the Mesolithic Period, 17 from the Early Neolithic, nine from the middle Neolithic, 18 from the Late Neolithic and 17 from the Final Neolithic (Table S1). DNA was extracted from teeth, long and petrous bones via phenol/chloroform extraction and concentrated by Amicon Ultra-15 centrifugation (Supplementary Information). Illumina sequencing libraries were prepared according to conventional protocols for ancient DNA^65^ using different indexing strategies for bone/tooth and petrous bone samples (Supplementary Information). Ancient DNA preservation was determined by quantitative Real-Time PCR and a shallow shotgun sequencing approach^3^. The mitochondrial genome was enriched by Agilent’s *SureSelect* Target enrichment (custom design) with adapted protocols for highly fragmented, deaminated and low copy number of endogenous mitochondrial DNA molecules. The enriched libraries were sequenced on Illumina platforms (MiSeq—50bpSE, 150bpSE; HiSeq 2500—100bpPE) targeting 1–2 million reads per sample. Blank controls were processed, sequenced and screened for contaminating molecules in each step of the protocol. Data analysis was performed as described elsewhere^3^ and tools to estimate the authenticity of ancient DNA sequence data were applied to the compiled dataset^66^. Several independent extractions of a sample were merged and contamination estimates^67^ were drawn from the combined dataset per sample (Table S2).

### Genetic diversity in Neolithic Greece

To explore the genetic diversity of the population from Neolithic Greece, we divided the initial dataset into three chronological groups: Early Neolithic (n = 17), Middle/Late Neolithic (n = 27) and Final Neolithic (n = 17). All sequences were cut to the HVS-I region at position 16.051–16.400 bp in the reference. The C-stretch polymorphism 16189C/T is excluded from the analysis. Arlequin 3.5^68^ was used to explore the molecular diversity within each population of the dataset, and compute indices of genetic differentiation (*Fst*) between pairs of samples using the Kimura P2 model to describe molecular distances between sequences. In our dataset we also included five previously published individuals: three from the Early Neolithic sites of Nea Nikomedeia (Nea2 and Nea3^69^) and Revenia (Rev5^3^) and two from the Final Neolithic sites of Paliambela (Pal7) and Kleitos (Klei10)^3^ (Table S1).

To visualize relations among samples, we conducted a multidimensional scaling analysis (MDS) using the R function isoMDS from the package MASS on our samples, complemented with a reference panel consisting of 751 ancient (dating from the Upper Palaeolithic to the Final Neolithic) and 1719 present-day individuals from southeastern and central Europe (Table S3).

### Spatially explicit simulation framework

To investigate population continuity in northern Greece and the relationship between Neolithic farmers along the Danubian expansion route from the northern Aegean to the Balkans and central Europe, we adapted the spatially explicit simulation framework initially designed by Currat and Excoffier^70^ and later improved by Silva et al.^71^ using a modified version of the program SPLATCHE2^72^. This framework allows the simulation of mitochondrial lineages at different points in time and space under alternative scenarios of population dynamics. Based on the two-layer spatially explicit model, we simulated two consecutive human expansions in a virtual European map divided into cells of 100 × 100 km (Fig. S1). Each cell is made up of two separate demes representing hunter-gatherers and farmers, respectively, resulting in two superimposed layers of demes over the whole map. The two superimposed populations in one cell compete and may admix, as described below. This modified version of SPLATCHE2 allows for varying admixture through time, varying competition through space, and a reduction of the local migration rate using the new parameter Mdec (see below).

The first layer, termed HG for hunter-gatherers, represents the expansion of a Palaeolithic population starting around 1600 generations ago (~ 40,000 years considering a generation time of 25 years) with 100 individuals from an arbitrary deme set in the Near East (P in Fig. S1, Table 1). The parameters used for the HG layer were fixed based on previous knowledge. They serve to fill the map with pre-Neolithic populations and no inferences were made for this layer. Each deme has a carrying capacity (*K*_*HG*_) of 100 effective haploid females, which corresponds to 200 individuals (males and females) per cell and a human density of 200*3/10,000 = 0.06 individuals/km^2^^73,74^, assuming that the census size was three times the effective population size^75^. The migration rate (*m*_*HG*_) and growth rate (*r*_*HG*_) were set to 0.15 and 0.2, respectively, to achieve a colonization of Europe in approximately 500 generations, following Silva et al.^71^ (Table 1). The *m*_*HG*_ represents the proportion of individuals in each deme emigrating to neighboring demes at each generation and the *r*_*HG*_ represent the intrinsic rate of population growth per generation.

**Table 1:** Input parameter for SPLATCHE2 for the various scenarios simulated.

Analysis	Scenario name	Paleo. layer	Neo. layer	*r*_*HG*_	*m*_*HG*_	*K*_*HG*_	*r*_*FA*_	*m*_*FA*_	*K*_*FA*_	γ	*Mdec*	Admixture model
Continuity in Greece	SPC	–	Yes	–	–	–	0.53	[0.3–0.5]	[100–2000]	–	1	–
Neolithic spread-Danubian expansion axis	SN1	Yes	Yes	0.2	0.15	100	0.53	0.4	[500–1000]	[0.0–0.4]	[1–20]	Constant in all cells
	SN2	Yes	Yes	0.2	0.15	100	0.53	0.4	[500–1000]	[0.0–0.4]	[1–20]	Increasing with time in all cells
	SN3	Yes	Yes	0.2	0.15	100	0.53	0.4	[500–1000]	[0.0–0.4]	[1–20]	Constant in central Europe only
	SN4	Yes	Yes	0.2	0.15	100	0.53	0.4	[500–1000]	[0.0–0.4]	[1–20]	Increasing with time in central Europe only

*SPC* corresponds to the parameter used for Structured Population Continuity test and SN1 to SN4 represent the four alternative scenarios of the Neolithic spread along the Danubian expansion axis. The parameters of interest were drawn from prior distributions to make inferences by estimating their posterior distributions using the ABC (see text). *HG* stands for the hunter-gatherers population layer and *FA* for the farmer population layer; *r*_*HG*_ and *r*_*FA*_ stand for the growth rate in hunter-gatherers and farmers, respectively; *m*_*HG*_ and *m*_*FA*_ for the migration rates; *K*_*HG*_ and *K*_*FA*_ for the carrying capacities; γ for the assimilation rate and *Mdec* for the factor of migration rate decrease in farmers when their carrying capacity reaches 90%.

The second layer, termed FA for farmers, represents the Neolithic and subsequent periods with a population expansion starting 400 generations ago (~ 10,000 years with an average human generation time of 25 years) from a deme set arbitrarily in eastern Anatolia with 100 individuals (N in Fig. S1). The initial carrying capacity of these demes (*K*_*FA*_) was estimated using a uniform prior distribution of U[500, 1000] individuals, the upper limit corresponding to the maximum density estimated for the Linear Pottery culture (LBK) of ~ 0.6 individuals/km^2^^76^. Eighty generations before the end of the simulations (~ 2000 years ago), the value of *K*_*FA*_ is set to 24,000 to reflect an increase in population density during the Roman period (to ~ 14.4 individuals/*km*^2^^76^). The migration rate (*m*_*FA*_) and growth rate (*r*_*FA*_) were set at to 0.4 and 0.53, respectively, in order to fit the dates of the advance of the Neolithic from eastern Anatolia to central Europe via Greece, following Silva et al.^71^ Table 1).

A Lotka-Volterra model of competition^77,78^ with density dependent coefficients of competition is used to mimic the progressive disappearance of the hunter-gatherer subsistence strategy and its replacement by the farming strategy^70^. Under this model, and thanks to a higher carrying capacity, farmers from the Neolithic deme have a competitive edge over hunter-gatherers from the Palaeolithic deme located in the same geographic cell. To cope with the long persistence of hunter-gatherers in central and northern Europe^1,79^, we modified the original model^71^ by lengthening the cohabitation period with farmers up to 224 generations (until 4400 years ago) in an area representing central Europe in our simulations (Fig. S1). This was done by setting the coefficients of competition to 0 in this area, then setting *K*_*HG*_ to 0 at generation 1424. This prolonged cohabitation period allows us to sample the hunter-gatherer layer at the time corresponding to the real samples from the analysed dataset, which otherwise would be impossible since the Palaeolithic deme would be empty at the sampling date.

Gene flow can occur from the Palaeolithic/Mesolithic to the Neolithic layer to represent hunter-gatherers who adopted farming or the birth of a child in the farming population with a parent from each of the two populations. The amount of hunter-gatherer gene flow toward the farming population is regulated by the assimilation rate γ, where γ = 0.0 indicates the absence of gene flow and γ = 1.0 full mixing; γ represents the proportion of contacts between hunter-gatherers to farmers within a deme that results in gene flow from hunter-gatherers to farmers at each generation (see^70^ for details). Therefore, this rate, which we estimate from the data, quantifies the relevance of cultural transmission during the Neolithic expansion: a value γ = 0.0 reflects a purely demic and γ = 1.0 a purely cultural diffusion of the Neolithic package.

To be able to reproduce both the interpopulational and intrapopulational patterns of mitochondrial diversity along the Danubian route, we extended the original model of Silva et al.^71^ with the additional parameter *Mdec*, which reflects the factor by which the Neolithic migration rate (*m*_*FA*_) is divided in a deme once 90% of the Neolithic carrying capacity (*K*_*FA*_) is reached in the same deme, and thus represents a reduction of mobility in farmers after their initial spread when its value is larger than 1. *Mdec* was fixed at 1.0 (no reduction in mobility) for the continuity tests (see below) but inferred from the data when investigating the Danubian route of Neolithisation (Table 1).

For each simulated demographic scenario, we simulated mitochondrial DNA sequences of 347 bp length under the coalescent as described in Currat and Excoffier^70^ using a mutation rate of 7.5 × 10^–6^ mutation/generation and a transversion rate of 0.9841^2^. The computation of statistics both in simulated and real data was done using the program Arlequin 3.5 and parameters of interest were inferred with ABC-GLM^80^ as implemented in ABCtoolbox2^81^ available at https://bitbucket.org/wegmannlab/abctoolbox.

### Simulation of the Neolithic spread along the Danube expansion axis

Τhe Neolithic spread along the Danubian expansion axis was simulated following four different scenarios (SN1-4) differing in the mode and tempo of the admixture between hunter-gatherers and farmers:

#### SN1: constant admixture along the Danubian expansion axis

This scenario represents an expansion of farmers from southeastern to central Europe with various proportions of hunter-gatherer contribution regulated by γ. Admixture between hunter-gatherers and farmers occurs all along the Danubian Neolithic expansion axis from Greece to central Europe at a rate constant over space and time.

#### SN2: admixture increasing with time along the Danubian expansion axis

This scenario represents a rapid dispersal of Neolithic farmers from Greece to central Europe in an early phase, and a later assimilation of local hunter-gatherers in a second phase. The admixture between hunter-gatherers and farmers occurs all along the Danubian Neolithic route from Greece to central Europe, but it increases linearly during the coexistence of both populations in the same cell till reaching its maximum (γ) at the end of the cohabitation period (i.e., after some time hunter-gatherers disappear due to competition with the farmers).

#### SN3: constant admixture only in central Europe

This scenario represents a fast migration wave of farmers from Greece to central Europe without admixture along the way. To investigate whether admixture between hunter-gatherers and farmers could have been limited in the southern part of the Danubian route, we tested a scenario where the admixture occurs only in central Europe (Fig. S1), regulated by γ.

#### SN4: increasing admixture only in central Europe

This scenario represents a fast migration of farmers from Greece to central Europe without admixture in an early phase, then an assimilation of local hunter-gatherers in a second phase but restricted to central Europe, contrary to SN2 where admixture occurs all along the Danubian route. It is similar to SN3, i.e., the admixture between hunter-gatherers and farmers occurs only in central Europe (Fig. S1), except that admixture increases linearly with time during the cohabitation period till reaching its maximum (γ) at the end of the coexistence of the two populations, similarly to SN2.

For each scenario, we performed 160,000 simulations with values for the three parameters of interest in our study γ, *K*_*FA*_ and *Mdec* drawn from prior distributions: γ [0–0.4]; *K*_*FA*_ [500–1000] and *Mdec* [1–20] (Table 1). We set the maximum value for γ to 0.4, at which the assimilation of hunter-gatherers into farming populations is at its maximum. The other parameters for the FA layer (*r*_*FA*_ and *m*_*FA*_) were fixed to fit the extremely rapid Neolithic spread from the Αegean area to central Europe.

#### Simulation of genetic diversity

**Figure 1:**
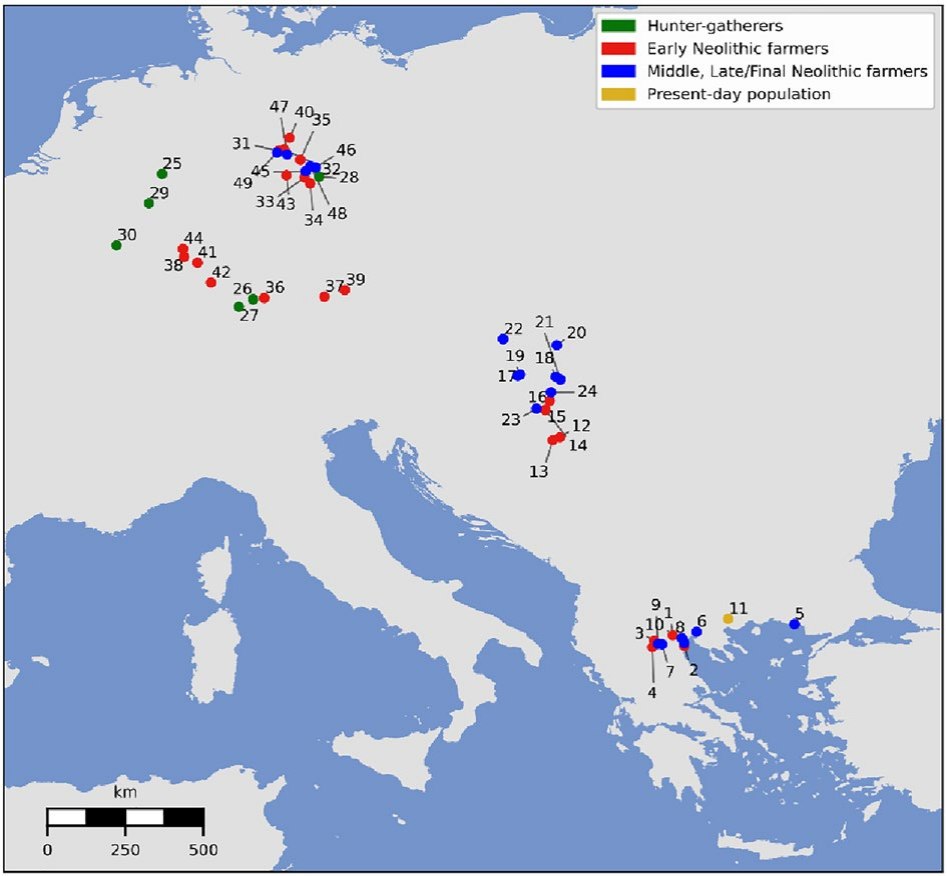
Geographical distribution of the mtDNA sequences (individual samples) used in the spatially explicit simulation framework. The coloured dots represent the geographical location of the mtDNA lineages from northern Greece (n = 45), central Europe (n = 200) and northern Balkans (n = 83). Hunter-gatherers (n = 19) are represented in green, Early Neolithic farmers (n = 177) in red, Middle and Late/Final Neolithic farmers (n = 132) in blue and present-day Greeks (n = 319) in yellow. Name (indicated in numbers) and precise chronology of the archaeological sites (locations) can be found in Table S4.

Each combination of parameters is used as input to SPLATCHE2 in order to generate genetic diversity in mtDNA sequences sampled at the precise geographic location and at the chronological date estimated for the real data available for Greece, the Balkans and central Europe (Fig. 1). We compiled 328 ancient sequences for this analysis, which we grouped into seven population samples according to their subsistence, chronological, and geographical characteristics: (1) hunter-gatherers from central Europe; Early Neolithic farmers from (2) Greece, (3) Hungary/Croatia and (4) central Europe; and Middle and Late/Final Neolithic farmers from (5) Greece, (6) Hungary and (7) central Europe (Table S4).

For each simulation, the genetic diversity was summarized in 14 statistics: the mean and standard deviation of the number of haplotypes (*k*), heterozygosity (*H*), mean pairwise differences (*π*) across all populations and of the *Fst* between each pair of (1) hunter-gatherers and Neolithic central European farmers (Early and Late); (2) Early Neolithic farmer samples (central Europe, Balkans and Greece); (3) Late Neolithic farmer samples (central Europe, Balkans and Greece) and (4) Early and Late Neolithic farmer samples from the same area (in central Europe, Balkans and Greece) (Table S5). All statistics were computed using the program Arlequin 3.5 under the Kimura P2 model^82^.

#### Model choice and parameter estimation

The ability of each model to reproduce the observed data was assessed with ABCtoolbox2 by computing a marginal density *P*-value ranging from 0 (no fit) to 1 (good fit). Model choice was then performed using two approaches. First, we calculated Bayes factors B_A_ in favour of scenario M_A_ over all other scenarios using ABCtoolsbox2. To validate this procedure, we used the cross-validation procedure available in ABCtoolbox2 that determines *PR*, the probability of recovering the correct model among the simulated data sets. Second, we used the model choice acceptance method of Pritchard et al*.*^83^, which assesses the relative fraction of each model among the best simulations (those with the smallest distances) among the combined set of simulations (here 640,000 simulations, 160,000 per scenario). We used ABCtoolbox2 to calculate distances and assessed the robustness of this approach by retaining the best 0.25%, 1% and 2.5% simulations.

We estimated the three varying parameters γ, *K*_*FA*_ and *Mdec* under the most likely model using the ABC-GLM^80^ method implemented in ABCtoolbox2. This method retains a small proportion δ of all *N* simulations based on the minimized Euclidean distance calculated between the simulated and observed statistics. The posterior distribution for each parameter is then obtained by approximating the truncated likelihood function using a general linear model (GLM). We set δ to 1.0% but also tested other fractions (0.25%, 2.5%) to ensure the robustness of the estimation.

Histograms representing the distribution of ‘posterior quantile’ and ‘posterior HDI’ for each parameter were plotted and used to test whether the posterior distribution of the parameters is biased compared to its prior distribution^84^. A total of 1,000 sets of statistics was generated under the best model with parameters drawn from the posterior distributions and considered as if they had been observed in reality (pseudo-observations). The positions of these true parameters are distributed uniformly in the marginal cumulative posterior distribution, if it is unbiased^85^. Deviation from the uniform distribution was detected by the Kolmogorov–Smirnov test. We also computed three different indices to evaluate the precision of our estimate: the relative bias (BIAS), the relative mean square error (RMSE) and the Factor2.

1 $$\mathrm{BIAS}=\frac{1}{n}{\sum }_{i=1}^{n}\frac{\widehat{|\theta }i-{\theta }_{i}|}{{\theta }_{i}}$$

2 $$\mathrm{RMSE} =\frac{1}{\overline{\theta }}\sqrt{\frac{1}{n}{\sum }_{i=1}^{n}{({\widehat{\theta }}_{i}-{\theta }_{i})}^{2}}$$

The Factor2 is defined as the proportion of *n* estimated values $\widehat{\theta i}$ lying in an interval bounded by 50% and 200% of their “true” value ${\theta }_{i}$. $\overline{\theta }$ is the average of ${\theta }_{i}$ over *n*.

### Testing population continuity in northern Greece

We used a Structured Population Continuity test (SPC) to investigate the relationship between mitochondrial lineages from different chronological phases in northern Greece. The aim was to assess whether populations from the earliest phases of the Neolithic can be considered directly ancestral to populations from the later Neolithic periods, but also to present-day populations from the same region. We thus grouped ancient mitochondrial sequences into two chronological phases corresponding to Early and Middle and to Late/Final Neolithic, thereafter, called serial population samples, and we compared them. For this we used the dataset of the 42 newly acquired mtDNA sequences excluding two Late/Final Neolithic samples from southern Greece, one from Franchthi Cave (Fra8) and one from Tharrounia (Tha2), due to their location in southern Greece, distant from the other sequences (Fig. 1, Table S4). To the 40 mtDNA sequences from northern Greece we added five already published mtDNA sequences^3,86^ as described above. We also compared those two Neolithic serial population samples with present-day mtDNA sequences from northern Greece for which we compiled a dataset of 319 mtDNA sequences^87^ (Fig. 1, Table S4).

The principle of this test, inspired by Bramanti et al*.*^2^, is to contrast the genetic differentiation (*Fst*) observed between samples from different chronological phases to that expected under a model of population continuity. As Silva et al*.*^88^ showed, population structure has a significant effect on the genetic differentiation between serial samples and, to get unbiased results, any such test must account for gene flow between subpopulations in the studied area. We thus first obtained ABC posterior samples of *Nm* values compatible with the observed genetic diversity among the samples. Specifically, we conducted 20,000 simulations under the SPC scenario (Table 1) with *Nm* values taken from 30 to 1000 using uniform distributions on the migration rate (*m*_*FA*_) and carrying capacity (*K*_*FA*_) centered on the values used for the simulation of the Danubian route and equal to [0.3–0.5] and [100–2000], respectively. We used larger priors to ensure a sufficient exploration of this important parameter for the population continuity test. Here, the Palaeolithic layer is only used to create the initial Neolithic population source. Each simulation was summarized with three statistics quantifying the intrapopulation diversity of the more ancient population sample among the pair under comparison: heterozygosity (*H*), pairwise differences (*π*) and number of segregating alleles (*k*). We then retained the 1,000 simulations closest to the observed statistics and confirmed their match with the observed data using the marginal density *P*-value outputted by ABCtoolbox2 (i.e., requesting *P* > 0.05).

The 1000 *Fst* values associated with the retained simulations constitute samples from the expected *Fst* distribution under a model of population continuity and with *Nm* values drawn from the ABC posterior. We used the proportion of those *Fst* values larger than that observed as a one-tail *P*-value to reject the model of continuity at the α = 5% threshold. If continuity is rejected, it suggests that the genetic differentiation observed between population samples cannot be explained by the stochastic processes of genetic drift, migration and sampling and hence an additional process should be invoked. Such an event could involve a population replacement (complete or partial) from a genetically distinct population occurring between the two sampling periods^88^.

### Ethics approval

We were given permission by the Greek Ministry of Culture and Sports to sample and extract DNA as well as to radiocarbon date all human remains mentioned in this study according to Greek law for destructive sampling of archaeological material (Ν.3028/02).

## Results

### Authenticity of ancient DNA results

The preservation state of the bone and tooth samples from the Mesolithic and Neolithic individuals was rather low. With few exceptions, the endogenous DNA content was below 0.5% (Table S2). Only the petrous bone samples showed higher DNA content.

Out of 70 samples screened, 42 mitochondrial genomes from Neolithic Greece were enriched and sequenced to a depth between 19 × and 300 × (Table S2). With the exception of St3A, Pal1 and Pal6, all results were replicated by two independent extractions. Samples Mau1, Mau2, and Krk2 were replicated by PCR and Sanger sequencing, which resulted in the same polymorphisms in the HVS-I region as determined by capture enrichment and NGS. The rate of post-mortem deamination at the first 5`position of DNA molecules ranged from 20 to 68% (mean 40%). DNA fragments averaged 86 bp (54–148 bp). The estimated fragment length obtained from 150 bp single end or 100 bp paired end runs shows a strong correlation with the deamination rate at the 3′position. Overall, 98.2% (6.6–99.9%) of all reads showed deamination patterns typical for highly degraded ancient DNA. Data showing signs of contamination (Mau1: 86.6% authentic data, Krk2: 94.4% % authentic data) were cleaned using PMDtools^64^ (Table S2).

### Descriptive statistics of diversity

**Figure 2:**
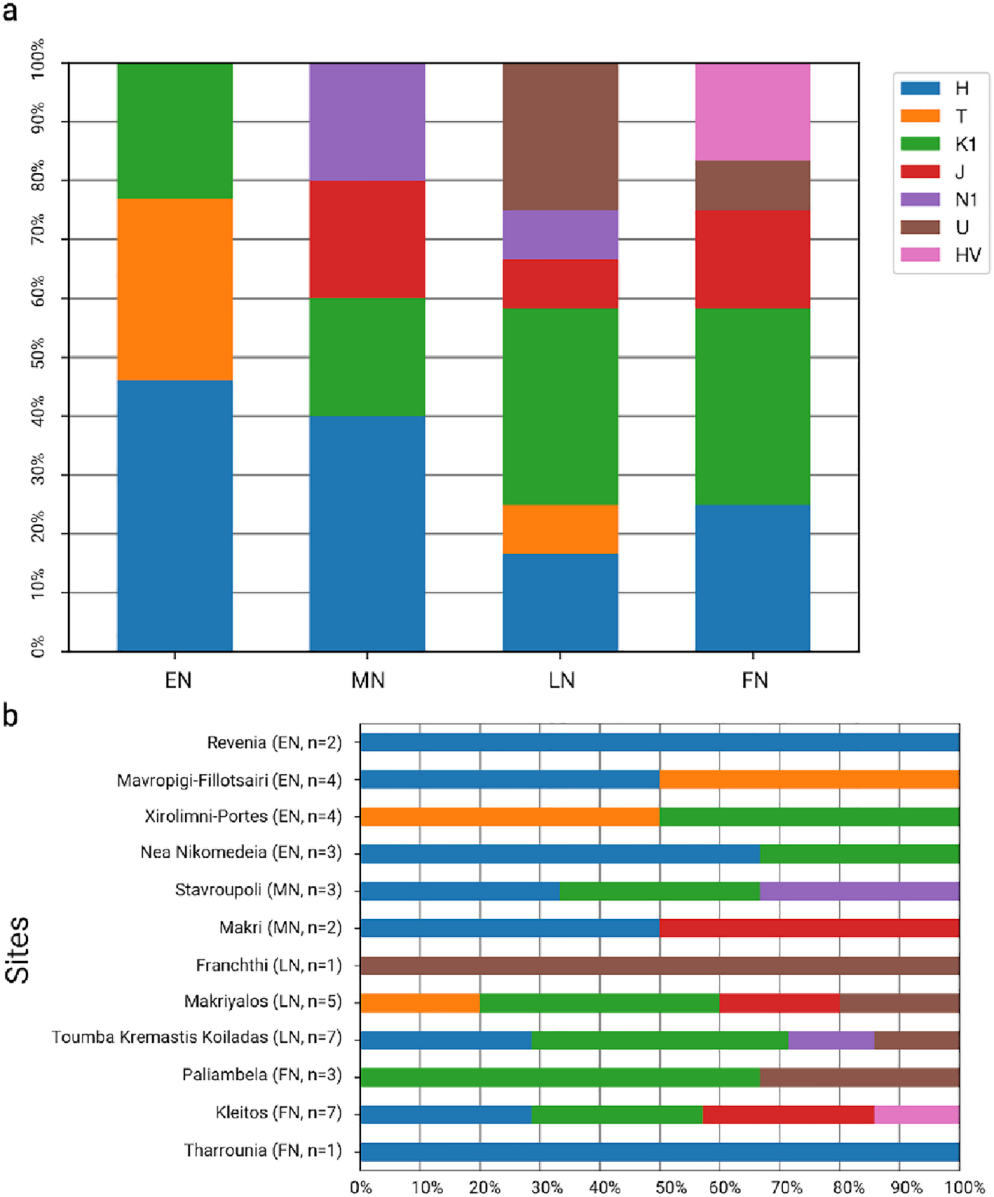
(**a**) Haplogroup frequency at different Neolithic periods in Greece. (**b**) Haplogroup frequency at different Neolithic sites in Greece (EN: Early Neolithic n = 13, MN: Middle Neolithic, n = 5, LN: Late Neolithic n = 13, FN: Final Neolithic n = 11).

The Mesolithic and Neolithic mitochondrial lineages from Greece correspond to the previously defined family of lineages—called haplogroups—H, T, K, J, N1, U, and HV (Fig. 2, Table S6). In our sample, the lineages belonging to Η and Κ have a frequency of 31% and 28.6%. Haplogroups T1 and T2 are observed predominantly during the EN, whereas the haplogroup J occurs from the MN onwards. Lineages belonging to haplogroup U* (U3, U4, U7 and U8) appear only during the LN and FN with a frequency of 9.5% while haplogroup HV is observed only during the FN. The entire dataset shows no U5 defining mutations (U5: position 3197, U5a: position 14793, U5b: 14182).

Gene diversity is at maximum and there is no difference between the EN, M/LN and the FN in Greece as all haplotypes are different within each sample (Ĥ = 1.0). Nucleotide diversity is low for all three Neolithic groups varying from π ~ 0.0114 ± 0.0067 for the Early, 0.0138 ± 0.0079 for the Middle/Late and 0.0089 ± 0.0056 for the Final Neolithic group.

The genetic distance between the EN and the M/LN (*Fst* = 0.0, P = 0.501) and FN group in Greece (*Fst* = 0.0, P = 0.419), as well as between the M/LN and the FN group (*Fst* = 0.0, P = 0.845), is not statistically significant, supporting an absence of genetic differentiation between those populations.

Differences on haplotypic variation between the Neolithic sites can be observed in Fig. 2a. The computation of pairwise *Fst* between archaeological sites show that most, are genetically undifferentiated (Table S7), in keeping with the very low sample sizes which demands caution in interpretation (2 < n < 8, Franchthi and Tharrounia were not included because they contain a single sequence). Among contemporaneous sites, EN Nea Nikomedeia (n = 5, Nea3: 6379–6091 cal BCE; Nea2: 6225–6075 cal BCE) shows significant genetic differences with Mavropigi-Fillotsairi (n = 4, Mau 16,333 ± 56 cal BCE). EN Mavropigi-Fillotsairi (n = 4) is also differentiated from LN Toumba Kremastis Koiladas (n = 7).

The genetic distance between the populations of the wider Aegean region (EN in northwestern Anatolia, EN, M/LN and FN in northern Greece) and the EN and MN in the Balkans and Carpathian Basin (Starcevo, Körös, Alföld-Linear Pottery phase I) is low and not statistically significant, supporting an absence of genetic differentiation between those populations. Significant *Fst* values > 0.045 (*P* < 0.05) can be measured in central Europe between the earlier and the final Neolithic periods (Table S8).

**Figure 3:**
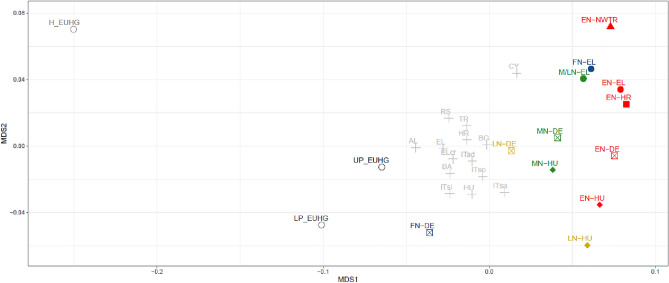
MDS with the 47 Greek Neolithic samples (42 newly sequenced and 5 published, EL, plain circles) and a reference panel of 26 ancient and present-day populations (2470 individuals in total), stress = 0.099 (EN: Early Neolithic, MN: Middle Neolithic, LN: Late Neolithic, FN: Final Neolithic, EUHG: hunter-gatherers, H_EUHG: Holocene EUHG, UP_EUHG: Upper Palaeolithic EUHG, LP_EUHG: Lower Palaeolithic EUHG, NWTR: north western Turkey, EL: Greece, DE: Germany, HR: Croatia, HU: Hungary, grey stars = modern populations abbreviations can be found on Table S3).

In a multidimensional scaling (MDS) analysis, based on *Fst* values calculated from the mitochondrial sequences (Table S8), all three Neolithic groups from present-day Greece fall close to each other (Fig. 3). The EN farmers from Greece plot on an axis connecting EN groups from northwestern Anatolia, Greece, the Balkans, and central Europe on the right of the graph. A similar axis is observed for the M/LN groups, among which the FN population from Greece also figures. On the contrary, FN central Europeans fall near the Palaeolithic and Mesolithic hunter-gatherers. Moreover, all M/LN and FN groups from Greece, Balkans, and central Europe shift to the left compared to their EN ancestors. Present-day populations from southeastern Europe and Hungary form a distinct group that falls between Palaeolithic and Mesolithic hunter-gatherers and Neolithic farmers.

### Structured population continuity test

The present-day inhabitants of northern Greece are significantly different from the Neolithic population from the same geographical area (*Fst* = 0.034, *P* = 0.002 between EN and present-day Greek populations and *Fst* = 0.039, *P* < 0.001 between ML/FN and present-day Greek populations). Even if the observed genetic differentiation is significant, it may be due solely to stochastic processes of sampling, local migration and genetic drift over time, without necessarily involving population turnover. The structured population continuity test checks this possibility by taking into account the spatiotemporal variance among sequences and it clearly rejects population continuity between the Neolithic sample and the present-day population (*P* = 0.010 between EN and modern and 0.012 between ΜL/FN and modern). In contrast, population continuity is not rejected between the earlier and later phases of the Neolithic (*P* = 0.338). The marginal density *P-values* are 0.44 and 0.60 for the simulations of mitochondrial diversity in EN and ML/FN, respectively, showing that the simulation framework is able to reproduce the empirical values and consequently that the structured population continuity test is valid in the current context.

### Neolithic spread along the Danube route

#### Estimation of the best scenario

The values presented in Table 2 show that all tested scenarios can reproduce the observed statistics (marginal density *P-*values > 0.58). The cross-validation shows that the probability of recovery is similar for all four scenarios (0.49 < PR < 0.54). Moreover, all results are robust to variation of the tolerance level δ.

**Table 2:** Model choice results.

	Tol. δ	Scenario SN1	Scenario SN2	Scenario SN3	Scenario SN4
Marginal density P	*0.25*	*0.893*	*0.970*	*0.868*	*0.798*
	1.00	0.858	0.936	0.877	0.584
	*2.50*	*0.869*	*0.845*	*0.873*	*0.441*
Posterior probability	*0.25*	*0.019*	*0.633*	*0.020*	*0.328*
	1.00	0.013	0.588	0.014	0.385
	*2.50*	*0.014*	*0.575*	*0.016*	*0.395*
Bayes factor	*0.25*	*0.019*	*1.727*	*0.021*	*0.487*
	1.00	0.013	1.426	0.015	0.626
	*2.50*	*0.014*	*1.351*	*0.016*	*0.653*
Probability of recovery	*0.25*	*0.442*	*0.440*	*0.442*	*0.517*
	1.00	0.540	0.493	0.509	0.522
	*2.50*	*0.466*	*0.468*	*0.490*	*0.523*

Marginal densities, posterior probabilities, Bayes factors (Model *i* against the others) and probability of recovery for each simulated scenario with a tolerance level of 1% (0.25% and 2.5% in italic).

We note that the two best scenarios are those where admixture between hunter-gatherers and farmers is increasing with time (posterior probabilities of SN2 = 58.8% and SN4 = 38.5% at δ = 1.0), whereas the two scenarios with constant admixture can be significantly rejected (SN1 and SN3 < 2% at any δ). Among the scenarios with the same admixture model, it was not possible to distinguish whether admixture occurred all along the Danubian route or only in central Europe (posterior probability of 48.1% for SN1 versus SN3 and 60.4% for SN2 versus SN4 at δ = 1.0).

#### Parameter estimation

**Figure 4:**
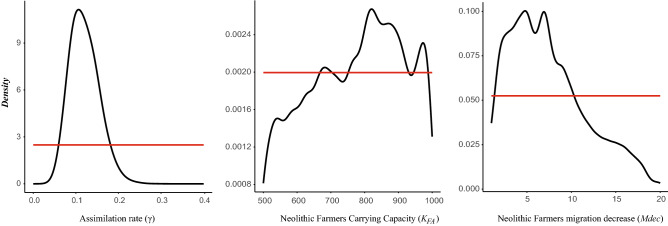
Prior (red line) and posterior (black line) distributions of the parameters estimated for the Neolithic expansion along the Danubian route under Scenario SN2. Τhe assimilation rate (γ) corresponding to the maximum gene flow from hunter-gatherer to the Neolithic farmer population, the carrying capacity of Neolithic farmers (K_FA_), the ratio of decrease of migration rate in Neolithic farmers after the colonization phase (Mdec).

For the best scenario SN2 (admixture increasing with time all along the Danubian route), we estimated γ at 0.107 (Highest Density Interval 90 = [0.062–0.176], Table 3, Fig. 4), the migration decrease *Mdec* at 4819 times (HDI 90 = [1.00–14.103]), and *K*_*FA*_ at 771 (HDI 90 = [539–974]). These point estimates reflect the mode for γ and *Mdec* and the mean for *K*_*FA*_, as we found those to be the most accurate on pseudo-observed data sets (Table 3).

**Table 3:** Parameter estimation results.

Parameters	Prior distribution	Tol. δ	Posterior distribution characteristics				Estimation precision		
			Mode	Mean	HDI 50	HDI 90	BIAS mode/mean	RMSE mode/mean	Factor 2 mode/mean
γ	0.0–0.4	*0.25*	*0.103*	*0.120*	*0.089–0.139*	*0.063–0.174*	*0.01/0.11*	*0.33/0.32*	*0.98/0.98*
		1.00	0.107	0.120	0.087*–*0.135	0.062*–*0.176	0.01/0.10	0.30/0.30	0.99/0.99
		*2.50*	*0.109*	*0.121*	*0.086–0.138*	*0.059–0.182*	0.02/0.13	0.33/0.33	0.98/0.97
Mdec	1–20	*0.25*	*6.729*	*7.620*	*2.289–7.576*	*1.00–14.005*	*0.26/0.51*	*0.57/0.48*	*0.68/0.77*
		1.00	4.819	7.501	2.269*–*7.636	1.00*–*14.103	0.23/0.50	0.58/0.49	0.69/0.77
		*2.50*	*4.915*	*7.662*	*1.907–7.558*	*1.00–14.222*	*0.32/0.51*	*0.55/0.47*	*0.71/0.77*
K_FA_	500–1000	*0.25*	*970*	*780*	*785–994*	*577–999*	*0.12/0.03*	*0.24/0.18*	*1.00/1.00*
		1.00	822	771	661*–*884	539*–*974	0.11/0.02	0.23/0.18	1.00/1.00
		*2.50*	*907*	*774*	*770–981*	*572–996*	*0.12/0.03*	*0.24/0.18*	*1.00/1.00*

Limits and characteristics of the prior and posterior distributions of the parameters estimated for the Neolithic expansion along the Danubian route under Scenario SN2 with a tolerance level δ of 1% (0.25% and 2.5% in italic). γ = assimilation rate between hunter-gatherer and farmer layer; Mdec = factor of migration decreases after reaching carrying capacity in farmers; KFA = carrying capacity of the farmer demes. HDI = Highest Density Interval. The precision of the mode and the mean of the posterior distributions are also given with three statistics (BIAS, RMSE and Factor2, see text for details).

To check if posterior estimates are unbiased, we inferred the distribution of quantiles and HDI of the true values among the posterior distributions on pseudo-observed data sets, which are expected to be uniform^84^. Uniformity was indeed rejected for γ (quantiles and HDI *P* < 0.001, Fig. S2), but not for *Mdec* (quantiles *P* = 0.131 and 0.863, Fig. S2). For *K*_*FA*_, uniformity was rejected for the quantiles at the 5% level (*P* = 0.030), but not for HDI (*P* = 0.472, Fig. S2). We thus consider the posterior estimates to be unbiased for *K*_*FA*_ and *Mdec,* but not for γ, which is slightly overestimated (Fig. S2).

## Discussion

### Haplotypic variation among ancient sites

Among the 42 ancient individuals newly reported here, we identified 7 different mtDNA haplogroups (K, H, HV, U, T, J and N1), all typical for populations of the Neolithic and highly related to the Neolithic expansion^67^.

As far as the distribution of mitochondrial haplogroups is concerned, the prehistoric individuals with haplogroup K in Greece are particularly interesting. Haplogroup K is a common lineage in our Neolithic sample. Previously, it has been used as a marker for the Neolithic expansion because it is virtually absent in Mesolithic hunter-gatherers from central and northern Europe^89^. However, lineages belonging to K1a have been recorded in two Mesolithic hunter-gatherers (Theo1, Theo5, ca. 7000 BCE) from the Theopetra Cave in Greece^3^ making them currently the only hunter-gatherers in Europe with a different haplogroup than U5. This lineage has been also found in pre-pottery Neolithic individuals from Israel^60^ and in central Anatolian Early Neolithic farmers (Boncuklu and Tepecik-Çiftlik^4^) signifying its presence in Anatolia and the Aegean even before the Neolithic expansion.

The entire dataset shows no U5 defining mutations, which is the predominant lineage of western European hunter-gatherers and is observed at high frequency in later phases of the central European Neolithic^9,62^ as well as in Mesolithic and Neolithic Italy^90^ and the Mesolithic Iron Gates^59^. This is remarkable, as it suggests that the local hunter-gatherer population in the Aegean and northern Greece may have been genetically distinct from those in the northern Balkans and other parts of Europe—as supported by the mitochondrial DNA evidence of Theopetra cave^3^.

Haplogroup N1a1a is one of the two lineages -the other being K1a- observed in Early Neolithic farmers of central Anatolia^4^. In Greece it is recorded only during the Middle and Late Neolithic Period.

Haplogroups N1, H, HV, J1, T1 and T2, have all been identified in Early Neolithic populations along the Danubian Neolithisation route *i.e.,* in present-day Serbia, Bulgaria, Hungary and Germany^7–9,63,91,92^.

### Genetic affinities between Neolithic sites in Greece, Anatolia and the Danube Neolithic expansion axis

Overall, we observe small differences in the frequency of haplogroups between Early, Middle, Late and Final Neolithic Greece. Consequently, genetic distances between these groups were low and not significant, supporting the scenario of a continuous, relatively homogeneous maternal population over the entire course of the Neolithic in Greece. In addition, most archaeological sites were not genetically differentiated, except for the site of Nea Nikomedeia that displays significant differences to EN Mavropigi-Fillotsairi. Although an intriguing finding especially in respect to the heterogenous and selective pattern observed in the material culture during the earliest phases of the Neolithic in Greece^54^, we refrain from making further inferences given the small sample size and our spatially explicit modelling results that indicate population continuity throughout the Neolithic. Interestingly, the remarkable increase in the number of settlements from the Middle Neolithic onwards^28,53^, the formation of more and larger communities that promoted more complex social structures and larger economic networks, were not triggered by a strong population turnover, at least not in the maternal line. Instead, endogenous population growth, possibly combined with increased local mobility, is likely to be the underlying phenomenon.

This is also supported by the low differences in gene and nucleotide diversity observed in the three Neolithic datasets from Greece especially when compared to the values of other Neolithic populations from the Balkans and central Europe^93^. Early Neolithic farmers from central Europe and Late Neolithic farmers from western Anatolia show similarly low gene diversity^4,8^. An overall increase of haplotype diversity from the Aegean to central Europe is only observed in early farmers along the Danubian route. This is in accordance with our results from the spatially explicit modelling that support an increasing gradient of hunter-gatherer contribution to the gene pool of farmers along the axis of the Danubian expansion, thus increasing genetic diversity by merging two differentiated genetic pools.

### Population continuity between Neolithic populations but discontinuity with present-day Greeks

The continuity between the Early and the Later Periods of the Neolithic in Greece has been verified by the application of an original test based on spatially explicit modelling, which considers ongoing local migration. This means that no fundamental population changes have taken place on the female side, but does not exclude minor and/or male migration. This changes during the very last phase of the Neolithic period. Previous work on two Final Neolithic (4500–4000 BCE) whole genomes from Greece, indicated gene-flow from populations with evidence of Caucasus hunter-gatherer-like ancestry^3,8^ that becomes stronger during the Early Bronze Age^94^. A similar trend is visible on the MDS plot (Fig. 3) as the Middle/Late and Final Neolithic groups shift away from the Early Neolithic groups but the difference is not big enough to result from population discontinuity according to the results of the structured population continuity test. Although we could not identify a significant external gene-flow for the maternal line, these signals could be related to the developments that took place in the Aegean at the end of the Neolithic. During the later phases of the Neolithic, maritime contacts and trade exchange intensified. The coastal zones and the islands were now populated and, on the mainland, certain settlements seem to have acquired considerable economic importance^95^, indicating that the more advanced economy of the following BA was unfolding.

On the contrary, present-day Greeks are descended not only from Neolithic Aegeans. Our results support population discontinuity between the Neolithic era and present-day Greece due to a genetically distinct population(s) immigrating to this region between those two periods. This observation is in line with the conclusion drawn from the analysis of three Neolithic genomes from this area for which DNA was retrieved at the genomic scale^3^. Thus, the results obtained for the maternal line are in accordance with those from biparental molecular markers. In this line of evidence, the recent analysis of six Early (3300–2000 BCE) and Middle (~ 2000 BCE) Bronze Age genomes from Greece showed that present-day northern-Greeks are genetically similar to 2000 BCE Aegeans from the same region^94^. Although they derive part of their ancestry from Neolithic farmers, a Neolithic Caucasus-like and BA Pontic-Caspian Steppe-like gene flow shaped the Aegean after the Neolithic period and may explain the population discontinuity we observe in our analyses.

### Neolithic spread along the Danube route

To trace the further spread of the Neolithic population from Greece to the Balkans and central Europe, we applied spatially explicit simulations and the ABC approach. We tested four competing scenarios, that consider different conditions of admixture between Neolithic farmers and hunter-gatherers: whether this happened everywhere in the simulation area or only in central Europe; and whether this occurred constantly or increased in intensity during the cohabitation period. The different scenarios were explored by varying three important parameters: (1) gene flow from hunter-gatherers to farmers, (2) the maximum farmer’s density and (3) the migration rate for farmers after their initial settlement. This initial period corresponds to the time it takes for a deme to reach 90% of its carrying capacity and it varies approximately between 200 and 400 years (8–16 generations) depending on the combination of parameters considered and the deme’s location. We found that the scenarios SN2 and SN4, under which admixture increases with time along the Danubian route, fitted the observed data much better than scenarios postulating constant admixture. In contrast, the data was inconclusive regarding the geographic area where admixture took place, i.e., whether hunter-gatherer assimilation occurred only in central Europe or also along the way from Greece. This difficulty in differentiating between scenarios with and without admixture in the first section of the Danubian route may be attributed, at least partly, to the fact that hunter-gatherer data is available only for central Europe. The fact that we modeled an extended cohabitation time in central Europe may also play a role because the resulting admixture is always higher in central Europe than elsewhere, independently of the scenario simulated.

Our results propose a cultural process by which hunter-gatherer ancestry has increased in European populations since the Middle Neolithic^62,63^, which did not occur: by accumulation through constant assimilation of hunter-gatherers into the farming community, but as the result of an increasing rate of assimilation, which did not take place early in the process, but only once farming was fully established. In our model, the rate of assimilation increases progressively throughout the duration of cohabitation between hunter-gatherers and farmers in each deme, which varies according to the combination of parameters and the scenario considered. It is generally of the order of 10 generations, but can go up to 100 generations in central Europe, i.e., roughly between 250 and 2500 years. Such dynamics correspond roughly to the ‘leapfrog’ colonization model^96,97^ with a rapid colonization of suitable niches for establishing agriculture followed by acculturation and genetic exchange with external non-farmers^98^. Under the most probable scenario, the assimilation rate γ was estimated at 11%. However, the HDI and quantile plots suggest that the point estimate for γ tends to be overestimated by about 20–25% (Table 3), and we thus conclude that along the Danubian route the proportion of contacts between hunter-gatherers and farmers that resulted in hunter-gatherer assimilation was probably around or slightly less than 10%. This is in line with the results of Silva et al.^71^, who estimated γ in central Europe to be ~ 2% from mtDNA (HDI90 = [0%–6%]) and ~ 9% from autosomal data (HDI90 = [5–14%]). The admixture rates estimated in the two studies cannot be directly compared, however, because they differ in the quantity they measure due to the different underlying admixture models (constant over time in Silva et al*.*^71^, but increasing over time in the current study). Our study therefore estimates a maximum admixture rate at the end of the cohabitation period (i.e., of a common land use), whereas Silva et al*.*^71^, estimated an average value during the same period, which is logically lower. In addition, our study includes a larger dataset covering the whole Danubian route, including the Balkans and Greece, whereas Silva et al*.*^71^, was geographically limited to central Europe.

Another important, but unexpected insight from the simulation study is that the high migration rate required to achieve a fast Neolithic spread between Greece and central Europe is only compatible with mitochondrial diversity if the migration rate decreases substantially after the original Neolithic settlement. In our simulation framework, it happens a few centuries after the colonization of a deme by farmers (i.e. after approximately 200–400 years), i.e., once 90% of the deme’s carrying capacity has been reached. We estimate that the dispersal rate of farmers must decrease by at least two-fold after the initial phase of a Neolithic settlement, and we estimate a five-fold reduction of the migration rate. This decrease of mobility after the phase of population growth that followed the initial Neolithic settlement (a few centuries in our simulation framework) corroborates anthropological observations^99^ as well as the estimation made from paleogenomic data with a different statistical approach^100^ and may be related to an increased sedentism^89^. This result is consistent with a gradual, almost plasmodic expansion of the Neolithic lifeway and the observation that each new settlement step was followed not only by a regional increase in population size, but also by pauses in expansion of up to several hundred years^12^.

Finally, the value estimated for the farmer’s carrying capacity points to the mean value of the range explored (modemean = 771, prior = [500, 1000]), which may be translated to a density of ~ 0.46 individuals/km^2^, lower than the maximum density of 0.6 individuals/km^2^ estimated for the Linear Pottery culture (LBK)^76^, but as the confidence interval is large, one should treat this estimate with some caution.

## Conclusion

We performed spatially explicit modelling with mitochondrial data whose resolution is lower than genomic multilocus data but which are abundant in an area from which whole genome data are sparse. Our results prove for the maternal lineage a homogeneous population inhabiting northern Greece throughout the Neolithic, without significant external gene flow until the end of this period. Our best fitting scenario suggests that Neolithic farmers expanded from the Aegean area to central Europe during a rapid migration event, with initially little admixture with local hunter-gatherers and increased gene flow over time. According to our results, the very high migration rate during the first phase of the Neolithisation process necessary in order to fit the temporal framework of the Neolithic spread must be followed by a substantial decrease in mobility to be compatible with the observed mitochondrial diversity. This phase of prolonged cohabitation may have been accompanied by an increasing local assimilation of hunter-gatherers and may explain the resurgence of hunter-gatherer ancestry from the Middle Neolithic onwards. In conclusion, the simulation approach presented here provides a solid framework for investigating the mechanisms of past population dynamics, which may be used for investigating ancestral genetic patterns in various spatio-temporal contexts of human migration and evolution.

## Supplementary Information

Supplementary Information 1.

Supplementary Tables.

## Data Availability

The accession number for the bam files of the ancient mtDNA genomes reported in study is European Nucleotide Archive: PRJEB52148 (https://www.ebi.ac.uk/ena/browser/view/PRJEB52148). The setting files and executable used for the simulation part of the paper can be accessed in the Zenodo public repository under: https://doi.org/10.5281/zenodo.6385610. Supplementary information to the present article, in addition to the supplemental tables and figures, are available in Document S1. The sources of the published sequences used for comparison with our newly produced sequences are provided in Tables S3 and S4.
