# Supplementary material for: Ancient mitochondrial diversity reveals population homogeneity in Neolithic Greece and identifies population dynamics along the Danubian expansion axis
Source: Sci Rep. 2022 Aug 5;12:13474. doi: 10.1038/s41598-022-16745-8 (PMC9356035; doi:10.1038/s41598-022-16745-8)
Supplement: Supplementary file 1 — Supplementary Information 1. [file 41598_2022_16745_MOESM1_ESM.docx]

# **Supplementary Information**

**Table of contents**

**1. Archaeological background of the analysed samples…………………………….… 4**

**2. Sample preparation………………………………………………………….…...……..17**

**3. Next-Generation sequencing methods………………………………………………..18**

**4. *Fig. S1:*** Digital map of Europe used for the simulation with SPLATCHE2…..……20

**5. *Fig. S2:*** Parameter estimation cross-validation………………………………………21

**6. References……………………………………………………………………………….22**

**Ancient mitochondrial diversity reveals population homogeneity in Neolithic Greece and identifies population dynamics along the Danubian expansion axis**

## Authors:

Nuno M. Silva^1 §^, Susanne Kreutzer^2 §^, Angelos Souleles^3^, Sevasti Triantaphyllou^4^, Kostas Kotsakis^4^, Dushka Urem-Kotsou^5^, Paul Halstead^6^, Nikos Efstratiou^4^, Stavros Kotsos^7^, Georgia Karamitrou-Mentessidi^8^, Fotini Adaktylou^9^, Areti Chondroyianni-Metoki^10^, Maria Pappa^11^, Christina Ziota^12^, Adamantios Sampson^13^, Anastasia Papathanasiou^14^, Karen Vitelli^15^, Tracey Cullen^16^, Nina Kyparissi-Apostolika^17^, Andrea Zeeb Lanz^18^, Joris Peters^19,20^, Jérémy Rio^1^, Daniel Wegmann^21,22^, Joachim Burger^2^°, Mathias Currat^1,23^°*, Christina Papageorgopoulou^3^°*

## Affiliations:

^1^ Department of Genetics & Evolution, University of Geneva, Switzerland.

^2^ Palaeogenetics Group, Institute of Organismic and Molecular Evolution (iomE), Johannes Gutenberg University of Mainz, 55099 Mainz, Germany

current address: Functional Genomics Center Zurich/GEML, ETH Zurich, Zurich, Switzerland & Department of Biology, ETH, Zurich, Switzerland.

^3^ Laboratory of Physical Anthropology, Department of History & Ethnology, Democritus University of Thrace, 69100 Komotini, Greece.

^4^ Faculty of Philosophy, School of History and Archaeology, Aristotle University of Thessaloniki, 54124 Thessaloniki, Greece

^5^ Department of History & Ethnology, Democritus University of Thrace, 69100 Komotini, Greece.

^6^ Emeritus, Department of Archaeology, University of Sheffield, Sheffield S1 3NJ, GB

^7^ Ephorate of Antiquities of Thessaloniki City, Hellenic Ministry of Culture and Sports, 54003 Thessaloniki, Greece

^8^ Ephor Emerita of Antiquities, Hellenic Ministry of Culture & Sports, 10682 Athens, Greece

^9^ Ephorate of Antiquities of Chalcidice and Mount Athos, Hellenic Ministry of Culture and Sports, 63100 Poligiros Chalcidice, Greece

^10^ Ephorate of Antiquities of Kozani, Hellenic Ministry of Culture and Sports, 50131 Kozani, Greece

^11^ Ephorate of Antiquities of Thessaloniki Region, Hellenic Ministry of Culture and Sports, 54646 Thessaloniki, Greece

^12^ Ephorate of Antiquities of Florina, Hellenic Ministry of Culture and Sports, 53100 Florina, Greece

^13^ Department of Mediterranean Studies, University of Aegean, 85132, Rhodes, Greece

^14^ Ephorate of Paleoanthropology and Speleology, Hellenic Ministry of Culture and Sports, 11636 Athens, Greece

^15^ Prof. Emerita, Department of Anthropology, Indiana University Bloomington, US; Director, Franchthi Cave Project

^16^ American School of Classical Studies at Athens, Princeton, NJ, US

^17^ Ephor Emerita of the Ephorate of Paleoanthropology and Speleology, Hellenic Ministry of Culture and Sports, 11636 Athens, Greece

^18^ General Direction for Cultural Heritage of Rhineland-Palatinate, Speyer, Germany

^19^ Institute of Palaeoanatomy, Domestication Research and the History of Veterinary Medicine, LMU Munich; Munich, Germany

^20^ SNSB, State Collection of Palaeoanatomy Munich; Munich, Germany

^21^ Department of Biology, University of Fribourg, 1700 Fribourg, Switzerland

^22^ Swiss Institute of Bioinformatics, 1700 Fribourg, Switzerland

^23^ Institute of Genetics and Genomics in Geneva (IGE3), University of Geneva, Switzerland

§ These authors contributed equally to this work

° These authors are joint senior authors on this work

* corresponding authors

## **Archaeological background of the sites analysed**

##

## **Greece**

## **Mesolithic Period**

#### Franchthi Cave

Franchthi cave is situated in the southern Argolid, on the eastern side of the Peloponnese on a rugged headland, across the bay from the modern Koilada village. The cave is situated about 12.50 m. above the modern sea level and overlooking the Paralia open-air Neolithic settlement. Excavations at Franchthi started in 1967, led by Thomas Jacobsen, as part of the investigations conducted by Indiana University and the University of Pennsylvania. Franchthi has proved to be a site of outstanding importance for the understanding of early Greek prehistory, as it was used over an extended period of time from the Upper Palaeolithic to the Final Neolithic and contains deep Mesolithic deposits^1–3^.

Dating based on ^14^C analysis gives a span for the Mesolithic strata from 9**,**016-8**,**192 cal 1σ BC to 7**,**242 BC-6**,**610 cal 1σ BC and for the Neolithic from 6**,**640-6**,**450 cal 1σ BC to 5**,**710-5**,**520 cal 1σ BC. Dating of findings outside the cave on the Paralia site span from 6**,**640-6**,**250 cal BC^4^. The Mesolithic and Neolithic strata inside the cave and on the Paralia have yielded finds including pottery, lithics, and faunal and botanical remains, as well as human burials. Important is the evidence for seafaring as early as the Palaeolithic, implied by obsidian from Melos, and in the Mesolithic, implied by numerous, large tuna vertebrae.

Samples Fra 1, Fra 2, Fra 3 belong to the Mesolithic layers and samples Fra 4-8 to the Neolithic layers. The individual Fra 8 is a young adult from trench FF1 from inside the cave. In the present study we analysed long bones from eight individuals, three Lower Mesolithic (Fr 1, Fr 2, Fr 3), two Middle Neolithic (Fr 4, Fra 5), one Late Neolithic (Fra 8), and two Final Neolithic (Fra 6, Fra 7) (Table S1).

#### Theopetra Cave

The cave of Theopetra is situated in the Thessalian plain on the north side of a limestone formation almost 100 m above the plain and 300 m above sea level. The cave lies between the edge of the plain and the eastern foothills of the Pindus mountains and is the westernmost prehistoric site of the Thessalian plain. The cave has a quadrilateral shape and measures approximately 500 m^2^. Excavations started in 1987 and, after fourteen seasons, ended in 2002. A second series of excavations was conducted from 2005 to 2008^5–7^. The archaeological remains are dated from the Middle Palaeolithic (130.6±5 ka) to the Late Neolithic, revealing a long sequence of deposits that extend across the Pleistocene-Holocene boundary, with the oldest dates ranging from 110-140 ka BP. The importance of the site lies in its Mesolithic layers that bridge the gap between the numerous Neolithic settlements of the Thessalian plain and open-air Palaeolithic findings. The samples analysed (Theo2, Theo3 and Theo4) belong to Mesolithic burials but did not yield sufficient mtDNA and therefore were excluded from further analysis. The samples Theo1 and Theo5 have been previously published^8^ (Table S1).

#### Maroulas

Maroulas in Kythnos is the only excavated Mesolithic site in the Aegean which has provided domestic remnants and burials during the 9^th^ millennium^9^. Kythnos is a Cycladic island close (ca. 40km) to the Greek mainland. The site of Maroulas is located near the coast close to the modern village of Loutra. Due to the rise of sea level, part of the settlement has been destroyed. Today the settlement measures 2000m^2^and mainly comprises circular or ellipsoid arrangements of flagstones that probably represent the floors of huts. In most of the cases human burials were found underneath the slabs. The burial types are rock-cut graves or cist graves comprising stone lined pits covered with large slabs. The lithic industry is characterized by adaptation to local raw materials primarily quartz and flint and secondly obsidian. Technologically the lithic industry is a flake technology. Compared to the Franchthi lithic industry of the same period, there are many dissimilarities in the morphological structure of retouched tools especially in the percentage of common tools. Terrestrial molluscs, of relatively few species, occur in enormous quantities over the entire extent of the settlement. Marine shells occur in smaller quantities while fish bones are also found. Animal bones were scanty especially from suids, small animals and birds. There was a complete absence of pottery. In the present study we analysed long bones from three individuals (Mar1, Mar2, Mar3) (Table S1).

## **Early Neolithic Period**

#### **Mavropigi-Fillotsairi**: An Early Neolithic settlement in western Macedonia (Greece)

The neolithic settlement of Mavropigi-Fillotsairi was excavated at the village of Mavropigi of the prefecture of Kozani^10,11^. The site was established on an almost flat area at 675 m altitude, on the western edge of the former marshland of Kitrini Limni, which is on the western edge of Ptolemaida between the Vermion and Askion Mountains. The site (app. 5000 m²) dates to Early Neolithic (6600-5900 BC) with three habitation phases^10,12–14^. Rescue excavations at Mavropigi-Fillotsairi were conducted in advance of the mining activities of the Hellenic Public Power Corporation (DEI) and revealed over 50 Early, Middle and Late Neolithic settlements ^11,15^.

The extensive rescue excavation revealed an Early Neolithic settlement with three major habitation phases (phase I, II, III). The main architectural feature of the Early Neolithic settlement is a large, oval pit (*central Orygma*) which was the first dwelling founded on the site, and remained in use throughout the occupation of the area ^10,13,16^. In the earlier phases (I and II), the *central Orygma* was a semi-subterranean structure, arranged in two rooms with clay floors, a round hearth and a main entrance. Two other prominent constructions, the *Ellipsoidal House* and the *western Orygma* with successive habitation floors have been dated to phase II. In the third phase (III) the *central Orygma* was replaced by a larger ground level structure with more complex plan. In this phase, in the area north and east of the *central Orygma*, seven (7) rectangular houses were recovered, constructed with wooden posts and clay floors.

The finds include pottery^17–19^, while the number of movable objects reaches several thousands. The lithic assemblage includes over 3000 chipped tools, made from local and imported materials and obsidian from Melos^20^,608 ground tools predominantly edged tools (picks and axes) and pounding and 18 interesting grinding tools^21^.Among the small finds, the excavations revealed loom weights, amulets, stone, bone and clay beads and six stamp seals of various shapes, bone pins, needles and some other crafted bones and horns^22^. From a total of 187 figurines, 184 were made of clay, two from stone and one from marble. Some of them exhibit new types, not found in other excavations in the region of Kozani^23,24^. The economy of the settlement was based on cultivation of domestic cereals and pulses (emmer, einkorn, barley, oats, lentils, peas or chickpeas) according to the archaeobotanical remains and on animal husbandry, as shown by the archaeozoological identification of 23 animal species^22^. Microfauna research did not identify remains of house mouse *(Mus musculus domesticus)* but only rodent species (*Mus macedonicus*/*spicilegus*)^25^. The results of herpetofauna are expected^22^.

In the excavated area, skeletal remains were found of 19 individuals in 17 graves (two of them contained a secondary burial). The deceased were placed in crouched position and were dated to Phase III. One more burial (number 13) was found in a supine position and was dated to the Byzantine period^26^. Child burials included seeds (burials 1 and 3^10,27^) and two of them were accompanied by “grave goods” (burials 7 and 9^10^). The human skeletal material has been studied to assess pathological conditions and dietary patterns (stable isotope analysis of human and fauna remains of the site)^26^. The results show that agriculture was already well established based on the absence of physiological and dietary stress in the population. Selected skeletons were also sampled for intestinal parasites^28^ and DNA analyses ^29^.

Absolute dating of charcoal, human and archaeobotanical samples, dates Phase I from 6,600 to 6,400 BC, Phase II from 6,400 to 6,300 BC and Phase III from 6,300/6,200 to 5,900 BC^10,11,16,17,22,24–27,30–32^. The settlement of Mavropigi-Fillotsairi is one of the Early Neolithic sites in Greece (and the Balkan peninsula) offering invaluable insight into the ongoing debate on the inception of the Neolithic in Europe.

Samples Mau1(burial no. 3) belongs to a 15-year old subadult individual, Mau2 (burial no. 5) belong to a 35-year-old woman ^26^. Direct ^14^C analysis of the Mau1 skeleton and dates this to 7,463±36BP/ /6,333±56 2σ cal BC.

In the present study we analysed teeth from two individuals (Mau1, Mau2) and long bones from two further individuals (Mau4, Mau5) (Table S1).

#### Xirolimni-Portes: An Early Neolithic settlement in western Macedonia (Greece)

The site Portes, close to the modern village of Xirolimni, was first discovered in 1993^33,34^. The archaeological site (officially named Xirolimni-Portes) is located on the narrow passage between the Vermion and Askion Mountains, that leads to western Greece. Extensive rescue excavations in three sectors began in advance of the construction of the national road Via Egnatia^14,33,35–40^.

Sector A included habitation remains dating mainly into the Early Neolithic period. The archaeological site, which extended beyond the Egnatia highway, was excavated in 5.000 m² and consists of deposits that reach 2 m in depth above the natural bedrock. The settlement was established on an almost flat area. A destruction layer was uncovered over a wide area, mainly consisting of building materials from ruined structures with stone bases and dense surfaces of concentrated masses of clay, which are evidently the remains of clay-built structures^14^. The clay-built structures belong to small hut dwellings made with unbaked bricks or piled clay, with or without stone bases, floors of compacted clay, and roofs of plaited straw and reeds coated with clay, and beams that would have held it up, as is attested by the existence of a considerable number of post-holes of varying sizes and at varying intervals apart. The settlement's main habitation phase may be dated to the Early Neolithic, as may the extensive destruction fill and more generally a fairly thick, brown, sandy clay fill with a high proportion of pottery and finds. It is certain that the Xirolimni-Portes settlement produced a very local type of pottery with some distinctive characteristics.

Most of the excavated objects (5,645 from a total of 9,443) date to the prehistoric period. The finds include lithic tools, chipped and ground stone tools, weaving objects and jewellery. Many objects, about 1,354, were made from bone (little were made from horn) and for several (288) it was possible to determine the species of the animal^22^. Very intriguing are the 57 figurines portraying standing and sitting anthropomorphic, birdlike and zoomorphic. They are all made from clay apart from a stone figurine resembling a frog, which was similar to another one found in Mavropigi-Fillotsairi as well as in Florina, Nea Nikomedeia and other sites in the Balkans^23,40^.

In the Neolithic settlement, 14 buried individuals were found in 12 graves (two contained a secondary burial) in a crouched position (all except for three) in pits, that were dug after the destruction layer. An established burial practice was the deposition of human remains in commingled assemblages. Many of the burials were intact with no accompanying grave goods. One skeleton was radiocarbon dated to 6,164+/- 51 BCE. The human skeletal material has been studied to assess pathological conditions and dietary patterns (stable isotope analysis of human and fauna remains of the site)^26^.The results show that the population was not under physiological and dietary stress, therefore it is assumed that the agriculture had already been established. Selected skeletons were also sampled for aDNA analyses^29^.

Among the faunal remains, 21,7% (23,613 identified from 108,557 remains) belong to domestic animals (sheep, goats, pigs, cattle and dogs) whereas lion and beaver were also found. Microfauna research did not identify remains of house mouse *(Mus musculus domesticus)* but only rodent species (*Mus macedonicus*/*spicilegus*)^25^. The results of herpetofauna are expected by Michalopoulou. Part of the ostreoarchaeological material has been studied by Theodoropoulou**.**

The Early Neolithic date of the settlement of Xirolimni-Portes is confirmed by both relative ceramic dating and absolute radiocarbon dating of samples (charcoal and bones) to 6210-6030 cal BP^11,14,26,35,41^. In the present study, five samples were analysed (Xir 1, Xir2, Xir3, Xir4B, Xir5). One sample (Xir5, burial Nr 10) was ^14^C dated together with samples from Mavropigi-Fillotsairi in the Oxford Radiocarbon Accelerator Unit and dates to 7314±37BP/ /6164±51 2σ cal BCE. We analysed teeth from four individuals (Xir1, Xir2, Xir4a,b) and a femur from one (Xir5) (Table S1).

#### Revenia

The open-air, flat-extended settlement at Revenia-Korinos lies in a small valley in lowland Pieria, central Macedonia, north Greece, about 31 m above present-day sea level, northwest of the modern village of Korinos and about 5 km from the current shoreline, and covers at least 4 ha, as the surface finds suggest. The rescue excavation conducted at the settlement during 2001-2004 revealed more than 100 pits, cut into the natural bedrock. The pits vary significantly in shape and dimensions, from 0.5 to more than 5 m diameter and up to 1.68 m surviving depth. Based on construction elements (*e.g.,* size, associated postholes, occasionally burnt clay remains of the superstructure and other features), a number of pits were characterized as subterranean or semi-subterranean pit dwellings, while others as possible working areas and refuse pits. It is suggested that the fills of most, if not all, of the pits were the result not of random discard but of structured deposition, a feature also observed spatially: pits filled mainly with pottery are located to the northwest, centre and southeast of the excavated area, while pits that lacked pottery but contained animal bones, and pits that contained very few finds are located to the northeast. There are two different habitation modes at Revenia: subterranean pit dwellings and above-ground, rectangular post-framed structures, which were built over the pits or close to them^42,43^. The pit dwellings represent the earlier habitation period, in which, on the base of pottery style, two chronological phases can be distinguished by comparison with the better-known Thessalian sequence: an earlier Early Neolithic phase (EEN) and a later Early Neolithic phase (LEN). The habitation period with the above-ground, rectangular post-framed structures is poorly preserved and dated only to the second phase (LEN) of the settlement^43–45^. Six primary articulated inhumations in a flexed position, five secondary disarticulated inhumations (bone accumulations) and twelve cases of scattered isolated bones were found within the investigated area. According to the anthropological evidence, both sexes and all age groups are represented, while women are over-presented to men (four women versus one man). Five out of eleven articulated and disarticulated burials belong to subadults while adults die over their thirty’s. As regards the levels of health status, the skeletal remains would suggest intense muscular strain due to everyday and heavy activities, high rates of malnutrition and poor diet during childhood and remarkable rates of tooth decay due to frequent consumption of soft and processed foodstuffs. Some of the burials were related with particular artefacts while two out of the six articulated inhumations yielded clear evidence of secondary manipulation of the dead body^46^. The well-defined contexts from Revenia have yielded rich assemblages of several categories of skilfully manufactured artifacts and also of ecofacts, which indicate a settlement where early agriculture^47^ and livestock management^48^ were practised. The inhabitants of Revenia communicated and interacted with other settlements, participating in exchange networks, as pottery analysis^45^ and the presence of obsidian in large quantities^49^ indicate. The radiocarbon results from the earlier habitation period of the site (*i.e.*, the pits) show that Revenia is one of the earliest Early Neolithic settlements in north Greece and the Aegean in general. The initial phase of habitation is dated at around 6,600/6,550 BC. Habitation intensified at around 6,460/6,430 BC, accompanied by a shift in pottery style. This phase lasts until 6,200/6,100 BC when the pit habitation mode is followed by above-ground, rectangular post-framed structures^50^.

In the present study we analysed samples from five individuals (sample Rev1-4, Rev6). One sample Rev5 was previously genomically (WGS) analysed and radiocarbon dated^8^ (Table S1).

#### Nea Nikomedeia

The Neolithic settlement of Nea Nikomedeia is a low tell, located on the western edges of Giannitsa plain (central Macedonia). Today the settlement is situated some 30km from the coast, but during its Neolithic occupation was much closer to the Thermaic Gulf^51^. The excavations were carried out in the early 1960s. Three habitation phases dated to the EN have been distinguished. The settlement was abandoned during the MN and inhabited again in the LN. Archaeobotanical^52^ and archaeozoological^53^ analysis again identifies the inhabitants of Nea Nikomedeia as farmers of crops (einkorn, emmer, barley, lentil) and livestock (sheep, goats, cattle and pigs). In total, 27 inhumations have been found with bodies placed in a pit in a flexed position^54,55^. On the present study we analysed petrous bones from three individuals (Nea1, Nea4, Nea5) two samples (Nea2, Nea3) have been previously genomically (WGS) analysed^56^ (Table S1).

### Middle Neolithic Period

#### Stavroupoli

The settlement is situated in the northwestern part of the modern city of Thessaloniki (central Macedonia). It is a flat-extended site (ca 10 ha) located on a natural low hill flanked by two ravines. Salvage archaeological excavations started in 1994. Approximately 3000 m^2^ have been investigated so far. Archaeological deposits vary in thickness from 1 m up to 4 m. Two main phases of occupation, Stavroupoli I and II, are clearly distinguished. The earlier is dated to the Middle Neolithic (MN) and the early phase of the Late Neolithic (LN), while Stavroupoli II to the late phase of the LN or to the Final Neolithic (FN). Six ^14^C dates place the beginning of the first occupational phase at 5839-5531 cal BCE^57^. On the basis of architectural remains and pottery Stavroupoli I phase is divided into two subphases. The earlier habitation subphase (Stavroupoli Ia) consists of pit-dwellings, circular or irregular in shape, up to 5 m in diameter. Hearths and ovens were situated inside and outside the houses^58,59^. Black burnished and to a lesser extent red burnished wares are the characteristic pottery of subphase Ia. The Stavroupoli Ib subphase consists of rectilinear, above-ground and free-standing houses with floors of compacted soil. Cooking facilities in this subphase were also found both inside and outside the houses^59,60^. Characteristic pottery of this subphase is black burnished and black topped with carinated or conical shape^61,62^. Stavroupoli II dwellings had, at least in part, stone walls and floors of compacted soil. Pottery from this phase differs significantly from the earlier ones. The majority of vessels are red in colour, often with black or dark coloured painted decoration. Polished and ground stone tools^63^, chipped stone tools^64,65^, bone tools^66,67^, clay figurines and a marble one, and jewellery of shells and clay are some of the finds unearthed. In addition to the scattered human bones, six burials and one cremation were uncovered.

In the present study we analysed a long bone from one individual (St1), a tooth and a long bone (femur) from a second individual (St2), a tooth and skull fragment from a third individual (St3) and petrous bones from two individuals (St4, St5) (Table S1).

#### Makri

The Neolithic settlement of Makri is a coastal site, located ten kilometers to the west of the city of Alexandroupolis (Thrace). The habitation spread over the low mound. The use of nearby cave in the same period is also attested. Systematic excavations were undertaken from 1988 to 1996 by the local Ephorate of Antiquities and the University of Thessaloniki^68^. Makri belongs to the tell type of sites with deposits that reach 4m in height. The settlement was thoroughly investigated by at least 16 trenches (each 25m^2^), located on the top, slope and periphery of the mound, covering an area of at least 2000 sq.m. The excavations revealed that the site was inhabited during the late EN-MN and LN, later prehistoric periods and historical times. The stratigraphic sequence of Neolithic Makri is divided into two main phases, Makri I and II, which are separated by a destruction layer and characterised by the changes in pottery typology^69^. The earlier Makri I phase, found only on the top of the mound, is characterized only by one habitation phase. The pottery from this phase, though sharing similarities with the later Makri II ceramics, differs from the latter in terms of decoration and, to a lesser extent, in vessel shapes. Makri II constitutes the main cultural period of the settlement. Deposits of this period were found on the top, slope and periphery of the mound. Four habitation phases have been distinguished. The Neolithic settlement covers a period between the end of the 7^th^ and the beginning of the 5^th^ millennium^70^. In the present study we analysed a maxillary molar from one (NS1) and a mandibular canine and right molar from another individual (NS2) (Table S1).

## **Late Neolithic Period**

#### Toumba Kremastis Koiladas

The site of Toumba Kremastis Koiladas is situated on the southeastern side of the Kitrini Limni basin, 20km north of the modern city of Kozani (western Macedonia). The site covers an area of almost 8ha. Salvage excavations in an area of almost 1 ha were conducted in 1998-99 in advance of construction of the Egnatia highway. Part of the NE boundary of the settlement was excavated and the finds confirm that this area was outside the residential space. The excavations revealed at least 462 pits ranging from 0,50 to 3,75 m in diameter and 0,10-2,90 m deep, and a series of 6 ditches. Several pits contained structured burials of animals, people, miniature pots, musical instruments (flutes), polished and ground stone tools and house models suggesting that this area on the margins of the settlement was related to ritual activities. Two main chronological phases have been distinguished. Human burials include 23 cremations and two inhumations with bodies in flexed position, all found inside the pits. The cremation burials are related to the last use of the excavated area. The large pits and one of the ditches may have been dug for the extraction of clay presumably used as building material for the construction of houses and perhaps for pot making. The burials were customarily cremated, representing a change in burial practice. This is the first cemetery identified in western Macedonia for this period. On the basis of pottery typology and a series of ^14^C determinations the site is dated to the early phase of the LN (5400-5000 BC)^71,72^. In the present study we analysed long bones from seven individuals (Krk1, Krk2, Krk3, Krk4, Krk5, Krk6, Krk7), teeth from two individuals (Krk8, Krk8) and petrous bones from two individuals (Krk10, Krk11) (Table S1).

#### Makriyalos

The Neolithic site of Makriyalos is a flat-extended site located on gentle hill slopes in the coastal lowlands of Pieria (central Macedonia). Rescue excavations, carried out in the 1990s, uncovered two distinct phases of occupation. Makriyalos I, dated by pottery to the early phase of the Late Neolithic (ca 5400-4900 BC), is represented by loosely scattered pit-houses. The settlement of this phase was encircled by two parallel ditches (Alpha and Beta), while part of a third (Gamma), found inside the settlement, must have separated part of it. At least 70 human burials, either intact or in the form of scattered human bones, were found in the settlement, many of them in the ditch Alpha. Makriyalos I has yielded exceptionally large artefactual and bioarchaeological assemblages. One of the most striking finds represents an unusually large pit exceeding 30m in diameter filled with the remains of large-scale feasting ^73^. This deposit yielded animal bones representing more than 600 domesticates, a huge volume of marine shells, archaeobotanical remains and numerous ceramic vessels used for cooking, serving and the consumption of food. Cups stand out from the rest not only in their (for the Neolithic) unusually large number, but also in their frequency of symbolically charged figurative handles. The Makriyalos II phase, which must have also been encircled by a ditch (or two parallel ditches), is represented by densely scattered pit dwellings, with foundations of at least one rectangular above-ground building also found. Among the interesting finds of this occupation phase is a group of hearths concentrated in one part of the settlement suggesting the preparation of food on a communal level. This phase is dated by pottery to the late phase of the LN (ca 4,900-4,500 BC)^74–76^. In the present study we analysed a tooth from one individual (Mkg1) and petrous bones from five individuals (Mkg9, Mkg10, Mkg11, Mkg12, Mkg13) (Table S1).

## **Final Neolithic Period**

#### Paliambela

The Neolithic settlement at Paliambela is situated in the rolling landscape of the coastal lowlands of Pieria in central Macedonia. An ongoing excavation of the site started in 2000 as a joint project between the Universities of Thessaloniki (Greece) and Sheffield (UK). The site is a low mound with Neolithic deposits exceeding 3m, spanning the period from the Early to the Final Neolithic. The total excavated area exceeds well over 500m^2^.

In the earlier phases of the Neolithic, it seems that Paliambela was a flat, extended site, while in the later phases the site took the form of a mound. It does not, however, represent a classic tell village of densely packed houses^77^. On the basis of the first preliminary reports, at least one deep ditch dug in natural bedrock, and a series of pits containing pottery, chipped stone and bone tools and other findings, belong to the Early Neolithic Period (6600-5900 BCE)^78^. During the Middle Neolithic (5,900-5,400 BCE) the settlement was also encircled by at least one deep, wide ditch. ‘Domestic’ architecture in this phase was comprised of closely-set rectangular buildings separated by cobbled yards. There is some evidence that habitation shifted across the site during the Middle Neolithic. Pottery from this phase links the settlement with both the southern Balkans and Thessaly ^44^.Late Neolithic [LN] (5400-4700 BCE) deposits found on top of the hill have been heavily eroded and disturbed by ploughing. It seems, however, that during the LN the settlement was encircled by a pair of stone enclosure walls ^79^. Based on the presence of pottery, the site was inhabited during the whole LN period up to the Final Neolithic (4700-3300 BC). Apart from black burnished and black topped vessels with characteristics of the LN period, carinated and conical shapes, and painted pottery of the ‘Dimini’ style is also found ^80^. Other findings include a large number of ground, polished, and chipped stone and bone tools, and figurines. Most of the human skeletal material belongs to scattered postcranial and a few cranial remains^81^.

The sample Paliambela 7 (Pal7) was submitted for radiocarbon dating at the Curt-Engelhorn-Zentrum of Archaeometry in Mannheim, Germany (Lab number MAMS 23037), yielding a ^14^C age of 5559 ± 29 BP and an estimated 95% calibrated range (based on OxCal v4.2.2 using INTCAL13) of 4452-4350 cal BCE. In the present study we analysed teeth from three individuals (Pal1, Pal2, Pal6) and one sample (Pal7) has been previously genomically (WGS) analysed ^8^.

#### Kleitos

The archaeological site of Kleitos is situated in western Macedonia, in northern Greece. In 2006, an extensive rescue excavation took place over an area of 7.5 hectares. This brought to light two neighboring Neolithic settlements (Kleitos 1 and 2) and findings of the Bronze Age, Hellenistic, Late Roman and Early Byzantine periods. Kleitos 1 is a flat Neolithic settlement inhabited during the early phases of the Late Neolithic period (second half of the 6th and early 5th millennium BC) and is one of very few sites in the Balkan region to have been excavated throughout its entire area^82^. Ten quadrangular ground-floor buildings made of a wooden framework and covered with clay have been identified. Inside the buildings were structures designed for food preparation and storage, along with clay vessels, tools, and pots. The buildings had been destroyed by fire and none of them preserved more than three construction phases. Among and around the buildings, a variety of workshops, storage areas, and refuse pits were uncovered. The settlement covers an area of approximately 2 ha and is bounded by a system of ditches and wooden enclosures. Inside Building C, amongst and outside the houses, 16 Neolithic graves were found. The burials were single inhumations in contracted positions and there was one cremation in an urn^82^. Sample Kleitos 10 (Klei10) was taken from a petrous bone. The sample was dated at the Curt-Engelhorn-Zentrum of Archaeometry (Mannheim, Germany). The results were calibrated as described above. For Klei10 (Lab number MAMS 23038) a ^14^C age of 5559 ± 22 BP was estimated resulting in a 95% calibrated range of 4230-3995 cal BCE. In the present study we analysed tooth and bone fragments from one individual (Klei1/Klei4), tooth alone from another (Klei2), the petrous bone and other bone fragments from another individual (Klei3/Klei9), bone fragments from two individuals (Klei5, Klei6), and petrous bones from two individuals (Klei7, Klei8)(Table S1), while one sample (Klei10) has been previously genomically (WGS) analysed^8^.

#### Tharrounia

**The cave of Skoteini at Tharrounia (Euboea island)**

The Skoteini cave is situated 3 km from Tharrounia village in the middle of the island of Euboea. It is situated just below a rocky plateau, 450 m above sea level. The cave opens to the north on to a ravine. The site was revealed firstly in 1974 by the discovery of ceramic sherds dated to the Neolithic^83,84^. Excavation began in 1986 and ended in 1991 revealing marble figurines and ceramics, metallic objects^85^, The Neolithic pottery was associated with three periods, specifically *the Middle Neolithic* (before the middle of 6^th^ millennium) resembling a pottery style also found in Nea Makri^86^, *the first part of the Late Neolithic* (5300-4800 BC) and the *Final Neolithic* (4300-3300 BC), which includes bigger vessels (pithoi) that signify the use of the cave for storage during this period. Skeletal remains were excavated in many layers of the cave.

On the plateau above the cave (named Plataki), archaeological research revealed settlement remains that date to the end of the Final Neolithic^85^ and a cemetery related to the settlement and the cave of Skoteini (approximately 400m from the cave). The graves were dug in the ground and were of semicircular or trapezoid shape. They were severely damaged by intense cultivation, but are typologically similar to those at Kephala on the Aegean island of Kea^87^. Most of the graves were not accompanied by grave goods, but Late Neolithic II pottery, obsidian tools, and stone vessels (grinding stones) were found in close proximity. The lack of grave offerings inside the graves is a custom of the Late Neolithic II period also observed at Yiali on Nissiros^88^ and Kephala on Kea.

In the cemetery of Tharrounia most of the buried individuals were aged between 35-40 years of age. Graves 3 and 5 were smaller and contained only crania, from secondary burials. Similar graves were found in Kalithies Cave on Rhodes^89^. The anthropological analysis revealed that the population was homogenous with a strange cranial morphology, while the majority of the adult population suffered from osteoarthritis and one skeleton bore stress marks related to intensive labour. The population consumed animal resources and had signs of dental attrition with no signs of caries^90^. In the cemetery of Tharrounia the majority of individuals were determined as female while the burials inside the cave of Skoteini belonged to younger individuals. In the present study we analysed long bones from three individuals (Tha2, Tha3, Tha4) and one petrous bone (Tha1) (Table S1).

**GERMANY**

## **Neolithic Period**

**Dillingen-Steinheim**

Located on a high terrace with thick loess soils, between the Danube and the Egau rivers, the LBK site of ‘Steinheim’ near Dillingen, Swabia, was excavated in 1987 by the Bavarian State Office for the Preservation of Monuments^91,92^. The site consists of a short-lived LBK settlement possibly enclosed by a ditch. Two groups of burials were reported for this site, a small burial cluster, comprising up to 27 early Neolithic graves, some distance away from the settlement^91^ and a group of regular graves within the ditch^93^. As such, formal inhumations in LBK ditches are common in southern Germany; they did not result from a massacre. The individual sampled (Dil16) comes from the ditch and represents an 11-13 year old, genetically male. The new radiocarbon date obtained from this skeleton (MAMS-46039: 6,200 ± 25 BP, 7,235-6,998 cal. BP at 2σ) confirms that the ditch was infilled during an ‘older LBK’ phase, ca. 7,150-7,050 BP^94^.

**Essenbach-Ammerbreite**

The LBK cemetery and settlement of ‘Ammerbreite’ in Essenbach, Landshut County, Lower Bavaria, was discovered in 1981 during construction work on the northwestern edge of the modern village. Subsequent excavations until 1986 by Henriette Brink-Kloke uncovered remains of LBK habitation and a concentration of 29 graves, ca. 40 m away, attributed to an advanced phase of the LBK. One additional burial (grave 7) was found inside a reused settlement pit in the direction of the cemetery^95^. Only a section of the cemetery could be excavated. Anthropological study of the skeletons identified 10 children or infants and 15 sub-adults or adults, including seven females and six males^95^. The individual sampled for this project (Ess7) was the child in grave 2, ca. 9-10 years old, buried in contracted position on the left side, with the head oriented to the northeast. This grave was one of the more richly furnished graves, containing also objects normally associated with adult males (graves 16 and 24), such as a stone axe and a complete vessel. The vessel contained the fragment of a perforated bone comb and the left, upper incisor tooth of the child^95^. No radiocarbon date is available. The objects deposited in the grave are typical of the LBK, though the comb suggests a ‘young’ or ‘late LBK’ date, possibly in the range 7050-6900 BP^93^.

**Otzing**

The LBK cemetery and settlement ‘Gartenäcker’ in Otzing, Deggendorf County, Lower Bavaria, was excavated in the years 1998-2000. The site revealed 67 Linear Pottery house foundations and numerous burials and pits. It produced one of the largest ancient Neolithic skeletal series in Bavaria, and double burials are unusually common. Settlement activity lasted for several centuries, starting at the latest during the Notenkopf phase (stage II) and continued until the most recent southern Bavarian LBK (stage IV). Altogether, Otzing witnessed three centuries of settlement between *c.* 7,250 and 6,850 BP. The individual sampled in this study (Otz 3, Grave B 29, feature 1620/1) is well preserved and discovered orienting NW-SE while facing SW. It is a complete skeleton lying sideways in a right stool position, the upper part of the body placed on the sole or on a thin layer of backfill, with the legs and left hand resting on yet another burial (B 30) deposited in the same feature. The individual’s sex could not be ascertained morphologically, its age at death was estimated 10-14 years. Grave goods are lacking, but material culture in the backfill would date this individual to the younger LBK (phase IVa)^94^.

**Sample Preparation**

As part of a screening process the samples were first inspected macroscopically. The samples were prepared for extraction by sawing and milling as described elsewhere^8,96^. In case of highly fragmented or porous material further processing of a sample was avoided. From samples that were further processed 200mg of powdered bones or teeth were incubated in 7ml lysis buffer (EDTA 0.5M, pH0.8 (Ambion/Applied Biosystems, Life technologies, Darmstadt, Germany), Proteinase K (18U/ul; Roche, Mannheim, Germany), N-laurylsarcosine (0.5%; Merck Millipore, Darmstadt, Germany) at 37°C on rocking shakers for 48 hours. The DNA was isolated via phenol/chloroform/isoamyl alcohol (25:24:1, Roth, Karlsruhe, Germany) extraction, then desalted by stepwise washes with 12 ml HPLC-water and concentrated to approximately 150-200ul using Amicon Ultra-15 Centrifugal Filter Units (Merck Millipore, Darmstadt, Germany). Centrifugation speed was set to 2500g to achieve a greater recovery of fragmented DNA molecules. To generate biological replicates every sample was extracted twice and blank controls were processed during milling and extraction steps.

#### HVS-I Sequencing

For parts of the samples the mitochondrial HVS-I region (position 16011 – 16409) was sequenced by PCR and Sanger sequencing. PCR primers, PCR conditions and sequencing strategy are described elsewhere^97^.

## **Next Generation Sequencing methods**

#### Library preparation and quantification (sample quality assessment)

All libraries were prepared following^98^ with modifications described in previous study^8^ while leaving ancient DNA extracts unrepaired. Since we expect distinct DNA quality in the sample material, bone and tooth samples were treated differently to petrous bone samples regarding PCR and indexing strategy. For all PCR reactions AmpliTaq Gold^®^ DNA polymerase (Applied Biosystems) was used to overcome Uracil residues during amplification and to conserve deamination patterns to determine authenticity of ancient DNA. PCR products were purified with MSB^®^ Spin PCRapace (Invitek, Stratec Molecular, Berlin, Germany).

DNA extracted from bone/tooth samples was amplified in two immediately consecutive PCR reactions (10 cycles each), using P7 indices only while keeping P5 adapter structure in its original form. In the second PCR reaction Herculase polymerase (Agilent) was used with primer pair IS7/IS6. Petrous bone samples were processed as described in previous study^8^.

Quantification of library concentration and fragmentation pattern was performed by Qubit^®^ Fluorometric quantitation (dsDNA HS assay, Invitrogen) and Bioanalyzer measurements (HS, Agilent Technologies). For quality assessment of single libraries of samples and blank controls, a quantitative real-time PCR was performed. With the results of a molecular screening described in previous study^8^ SI 2), we selected samples for mitochondrial capture. Samples with low unique copy numbers and endogenous DNA contents were discarded, since previous experiments have shown high duplication and contamination levels in samples of similar quality (data not shown).

#### Enrichment of the mitochondrial genome and Illumina sequencing

All post-library-amplification experiments and hybridization reactions were carried out in a specific laboratory that is exclusively used for NGS experiments. Working steps were performed in UV-irradiated boxes. To avoid cross-contamination all objects of general use were incubated with bleach and UV-irradiated after usage. Capture design and setup is described in (^8^, SI 2). All samples were enriched for the mitochondrial genome in two serial capture rounds. For bone and tooth samples pooled libraries of two extractions were used, where library molecules carried P7 indices only; distinct extractions of a sample were indexed independently to guarantee a separate data analysis. P5-Indices were added during PCR after the second capture. On the contrary, we used just a tiny subset of the library for PP samples, with both indices added (see difference in PCR strategy).

Capture products were sequenced in a pool using different Illumina sequencing platforms and chemistry (MiSeq - 50bp SE, 150bp SE; HiSeq 2500 - 100bpPE). Illumina sequencing data analysis was performed as described in previous study^8^, SI 3). We used ContamMix^99^ to estimate contamination rates and display the authenticity of the dataset (Table S2). As a threshold for implementation of the data in subsequent population genetic analysis and modelling, we set the percentage of authentic data to 95% for each dataset. Additionally, each dataset per sample was filtered for deaminated reads (PMTtools^100^). SNP Call and contamination estimates were drawn from the filtered datasets in order to confirm the mitochondrial DNA lineage and sample authenticity.

## ***Supplementary Tables and Figures***

Table S1: All samples analyzed in this study (archaeological site, provenance, chronology, radiocarbon dates, sample type, archaeological ID)

Table S2: Screening results of all analyzed samples

Table S3: Reference dataset for the MDS

Table S4: Characteristics of the dataset used for the simulations

Table S5: Empirical statistics used for the ABC estimation

Table S6: mtDNA Haplogroups list of the newly sequenced samples from Greek Neolithic sites

Table S7: *Fst* values between Greek Neolithic sites

Table S8: *Fst* values used for the MDS plot between newly sequenced Greek Neolithic samples and reference panel

Figure S1: Digital map of Europe used for the simulation

Figure S2: Parameter estimation cross-validation

**Figure S1:** Digital map of Europe used for the simulation with SPLATCHE2. The dark grey square represents the central European area with a longer cohabitation period between hunter-gatherers and farmers. White cells represent water; light grey cells are not used for the simulations (islands); P and N represent the point of departure for the population expansion in the Paleolithic and Neolithic layers, respectively.


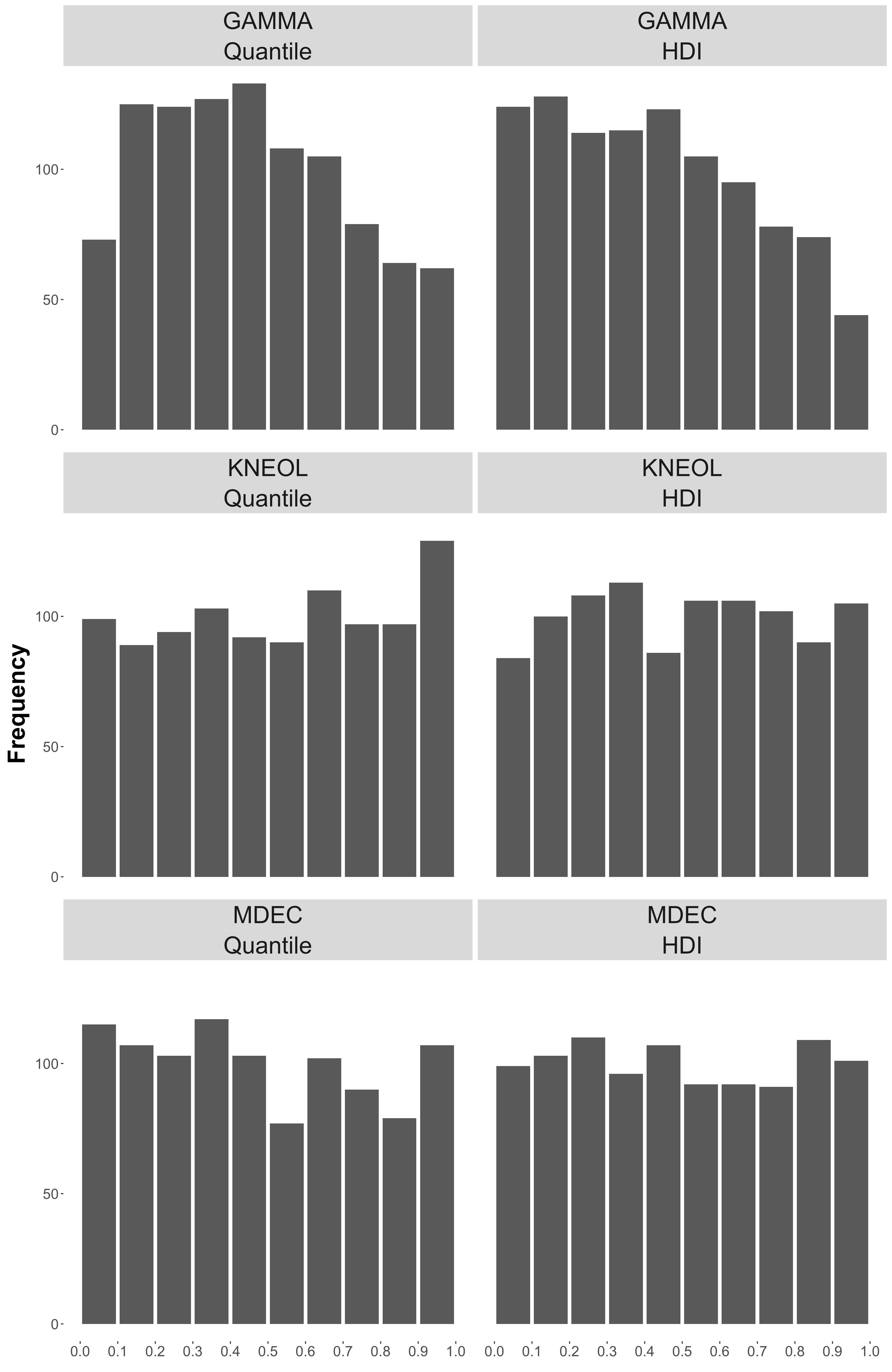


c

b

a

**Figure S2:** Parameter estimation cross-validation. Those histograms represent the distributions of HDI and quantiles for the three parameters estimated with the simulation of the Danube Neolithic expansion: A) parameter γ, B) parameter K*_FA_* NEO C) parameter Mdec. (γ = assimilation rate between hunter-gatherer and farmer layer; Mdec = factor of migration decreases after reaching carrying capacity in farmers; K_FA_ = carrying capacity of the farmer demes. HDI = Highest Density Interval).

**References**

1. Jacobsen, T. W., Farrand, W. R., Cooper, F. A. & Vitaliano, C. J. *Franchthi Cave and Paralia: maps, plans, and sections.* (Indiana University Press, 1987).

2. Jacobsen, T. W. Excavations in the Franchthi Cave, 1969-1971. Part II. *Hesperia* **42**, 253 (1973).

3. Asouti, E., Ntinou, M. & Kabukcu, C. The impact of environmental change on Palaeolithic and Mesolithic plant use and the transition to agriculture at Franchthi Cave, Greece. *PLoS One* **13**, e0207805 (2018).

4. Perlès, C. *The Early Neolithic in Greece: The first farming communities*. (Cambridge World Archaeology, 2003).

5. Kyparissi-Apostolika, N. The Palaeolithic deposits of Theopetra Cave in Thessaly (Greece). in *The Palaeolithic archaeology of Greece and adjacent areas: Proceedings of the ICOPAG Conference, Ioannina, September 1994* 232–239 (British School at Athens Studies, 1999).

6. Kyparissi-Apostolika, N. The Mesolithic/Neolithic transition in Greece as evidenced by the data at Theopetra cave in Thessaly. *Doc. Praehist.* **27**, 133–140 (2000).

7. Valladas, H. *et al.* TL age-estimates for the Middle Palaeolithic layers at Theopetra cave (Greece). *Quat. Geochronol.* **2**, 303–308 (2007).

8. Hofmanová, Z. *et al.* Early farmers from across Europe directly descended from Neolithic Aegeans. *Proc. Natl. Acad. Sci.* **113**, 6886–6891 (2016).

9. Sampson, A., Kaczanowska, M. & Kozłowski, J. K. *Τhe prehistory of the island of Kythnos and the Mesolithic settlement at Maroulas*. (Polish Academy of Sciences and Arts and University of the Aegean, 2010).

10. Karamitrou-Mentessidi, G. Mavropiyi 2005. Lignite mines and Antiquities. in *AEMTh 19* (eds. Adam-Veleni, P. & Tsakalou, K.) 511–539 (Ministry of Culture, 2005).

11. Karamitrou-Mentessidi, G. About prehistoric sites in western Macedonia: prefectures of Kozani and Grevena. in *A century of research in prehistoric Macedonia 1912-2012* (eds. Stephani, E., Merousis, N. & Dimoula, A.) 223–250 (2014).

12. Karamitrou-Mentessidi, G. L΄ Eforeia Proistorikon kai Klassikon Archaiotiton (Mavropigi, Fillotsairi site). *Archaiologikon Delt.* **60**, 763-816 (771–782) (2005).

13. Karamitrou-Mentessidi, G. *et al.* New evidence on the beginning of farming in Greece: The Early Neolithic settlement of Mavropigi in western Macedonia (Greece). *Antiquity* **87**, (2013).

14. Karamitrou-Mentessidi, G. Aiani and the Prefecture of Kozani: ten years of survey. in *AEMTH 20 years* (eds. Adam-Veleni, P. & Tzanavari, K.) 105–126 (Ministry of Culture, 2009).

15. Karamitrou-Mentessidi, G. Prehistoric settlements of Kitrini Limni. in *Amitos: Honorary volume for Prof. Manolis Andronikos* 391–416 (1986).

16. Karamitrou-Mentessidi, G., Efstratiou, N., Kaczanowska, M. & Kozłowski, J. Early Neolithic Settlement of Mavropigi in Western Greek Macedonia. *Eurasian Prehistory* **12**, 47–116 (2015).

17. Bonga, L. Pioneering potters: Early Neolithic ceramics from Mavropigi-Fillotsairi, Western Macedonia. in *Athens University Review of Archaeology* 9–62 (2020).

18. Bonga, L. Early ceramic styles and technologies in the Aegean and the Balkans: *Doc. Praehist.* **46**, 158–172 (2019).

19. Bonga, L. A. Thoughts on the Preliminary Study of Early Neolithic Decorated Pottery from the Central Origma at Mavropigi-Filotsairi. in *Communities, Landscapes, and Interaction in Neolithic Greece: proceedings of the international conference, Rethymno 29-30 May, 2015* (eds. Sarris, A., Kalogiropoulou, E., Kalayci, T. & Karimali, L.) 374–387 (International Monographs in Prehistory, Archaeological Series 20, 2017). doi:10.2307/j.ctvw049k3.31.

20. Kaczanowska, M. & Kozłowski, J. Early Neolithic Settlement of Mavropigi in Western Greek Macedonia. *Eurasian Prehistory* **12**, 71–116 (2015).

21. Ninou, I., Chondrou, D., Kasapidou, G., Karamitrou-Mentessidi, G. & Valamoti, S.-M. Does a mortar make a house…? Early Neolithic grinding and pounding stone tools from Greece: Mavropigi-Fillotsairi of Kozani (West Macedonia). in *Early Neolithic of Europe, 6-8 November* in press (CSIC and Maritim Museum of Barcelona, 2019).

22. Michalopoulou, S. Early Neolithic in Western Macedonia, Greece: the contribution of the zooarchaeological study from the sites Fyllotsairi -Mavropigi and Portes - Ksirolimni. (University of Athens, 2017).

23. Karamitrou-Mentessidi, G. Aiani. A coroplastic workshop of the Archaic era. in *Figurines. A microcosmos of clay* 64–67 (2017).

24. Starnini, E. The figurines from the Early Neolithic settlement of Mavropigi (Western Macedonia-Greece) and their significance in the neolithization process of Greece. *Eurasian Prehistory* **14**, 55–152 (2018).

25. Cucchi, T. *et al.* Tracking the Near Eastern origins and European dispersal of the western house mouse. *Sci. Rep.* **10**, 8276 (2020).

26. Papathanasiou, A. & Richards, M. P. Anthropologika kataloipa apo tis proimes theseis Mavropigis, Ksirolimnis kai Pontokomis tis Archaioteris Neolithikis sti Ditiki Makedonia. in *AEAM 1* 257–274 (2009).

27. Valamoti, S. M. Seeds for the dead ? Archaeobotanical remains from Mavropigi near Kozani, site Fyllotsairi. in *AEAM 1* 245–256 (2009).

28. Anastasiou, E. The Paleoparasitology of Eastern Mediterranean and Adjacent Regions: Understanding Intestinal Diseases Through Time. (University of Cambridge, 2013).

29. Papageorgopoulou, C. Ancient DNA: applications, perspectives, limitations. in *100 Years Research in prehistoric Macedonia 1912-2012* 477–491 (2014).

30. Maniatis, Y. Radiocarbon dating of the major cultural phases in prehistoric Macedonia: Recent developments. in *A century of research in prehistoric Macedonia 1912-2012 Research in prehistoric Macedonia 1912-2012* (eds. Stefani, E., Merousis, N. & Dimoula, A.) 205–222 (2014).

31. Douka, K., Efstratiou, N., Moundrea-Agrafioti, Α. & Karamitrou-Mentessidi, G. Dating the Early Neolithic of mainland of Greece, with a focus on Thessaly and Macedonia. in *Early Neolithic of Europe, 6-8 November* (CSIC and Maritim Museum of Barcelona, 2019).

32. Karamitrou-Mentessidi, G. *et al.* New evidence on the beginning of farming in Greece: the Early Neolithic settlement of Mavropigi in western Macedonia (Greece). *Antiquity* **87**, 1–4 (2013).

33. Karamitrou-Mentessidi, G. Voion-Notia Orestis: archaeologiki ereuna kai historiki topographia. in 104, 108, 212–215 (1999).

34. Karamitrou-Mentessidi, G. Xirolimni. in *Archaeologikon Deltion* vol. 48 380–381 (Ministry of Culture, 1993).

35. Karamitrou-Mentessidi, G. Xirolimni-Thesi Porta. in *Archaeologikon Deltion* 671–673 (Ministry of Culture, 1998).

36. Karamitrou-Mentessidi, G. Xirolimni Kozanis 1998. in *AEMTh 12* (ed. Adam-Veleni, P.) 465–480 (Ministry of Culture, 1998).

37. Karamitrou-Mentessidi, G. Xirolimni. in *Archaeologikon Deltion* vol. 54 629–632 (Ministry of Culture, 1999).

38. Karamitrou-Mentessidi, G. Xirolimni. in *Archaeologikon Deltion* vol. 55 794–796 (Ministry of Culture, 2000).

39. Karamitrou-Mentessidi, G. Prefecture of Kozani. *Archaeologikon Deltion* vols 56–59 379 (2001).

40. Karamitrou-Mentessidi, G. O Nomos Kozanis stin Proistoriki, Archaici-Classiki, Hellenistiki kai Romaiki Epochi. in *Kozani kai Grevena, O Choros kai oi Anthropoi* 249–256, 270–282 (2004).

41. Karamitrou-Mentessidi, G. Prefecture of Kozani 2000: Excavations on roads and by the wayside. in *AEMTh 14* (ed. Adam-Veleni, P.) 607–640 (Ministry of Culture, 2000).

42. Besios, M. & Adaktylou, F. The Neolithic settlement at “Revenia” Korinou (in Greek). in *AEMTh 18* (eds. Adam-Veleni, P. & Tzanavari, K.) 357–366 (Ministry of Culture, 2006).

43. Adaktylou, F. The Neolithic settlement at Revenia, Korinos, Pieria (in Greek) (Vol. I & II). (Aristotle University of Thessaloniki, 2017).

44. Urem-Kotsou, D., Papaioannou, A., Papadakou, T., Saridaki, N. & Intze, Z. Pottery and stylistic boundaries. Early and middle neolithic pottery in Macedonia. in *A century of research in prehistoric Macedonia 1912-2012* (eds. Stefani, E., Merousis, N. & Dimoula, A.) 505–517 (2014).

45. Urem-Kotsou, D. *et al.* Patterns in Contemporaneous Ceramic Traditions. in *Communities, Landscapes, and Interaction in Neolithic Greece* 324–338 (Berghahn Books, 2017). doi:10.2307/j.ctvw049k3.28.

46. Triantaphyllou, S. & Adaktylou, F. The manipulation of the deceased during the earlier phases of the Neolithic period in Macedonia: first remarks from Revenia, Korinos, northern Pieria (in Greek). in *AEMTh 28* (eds. Adam-Veleni, P., Arvanitaki, A. & Zografou, E.) 215–222 (Ministry of Culture, 2019).

47. Kotzamani, G. From gathering to cultivation: an archaeobotanical investigation of the early stages of plant exploitation and the beginnings of agriculture in Greece (Theopetra cave, Schisto cave, Sidari, Revenia) (in Greek). *PhD Thesis* (Aristotle University of Thessaloniki, 2009).

48. Isaakidou, V., Halstead, P. & Adaktylou, F. Animal carcass processing, cooking and consumption at Early Neolithic Revenia-Korinou, northern Greece. *Quat. Int.* **496**, 108–126 (2018).

49. Dogiama, L. Casting a Wide Network: Preliminary Results from the Early Neolithic Chipped Stone Assemblages from Revenia, Pieria (Greece). in *Communities, Landscapes, and Interaction in Neolithic Greece. Proceedings of the International Conference. Rethymno 29-30 May, 2015* (eds. Sarris, A., Kalogiropoulou, E., Kalayci, Y. & Karimali, L.) 446–462 (International Monographs in Prehistory. Archaeological Series 20, 2017).

50. Maniatis, Y. & Adaktylou, F. Revenia-Korinos: one of the earliest neolithic settlements in north Greece as evidenced by radiocarbon dating. *Radiocarbon* **63**, 1025–1051 (2021).

51. Ghilardi, M. *et al.* Human occupation and geomorphological evolution of the Thessaloniki Plain (Greece) since mid Holocene. *J. Archaeol. Sci.* **35**, 111–125 (2008).

52. van Zeist, W. & Bottema, S. *PALYNOLOGICAL INVESTIGATIONS IN WESTERN IRAN*. (1971).

53. Higgs, E. S. Fauna. in *Excavations at the Early Neolithic site at Nea Nikomedeia, Greek Macedonia* 271–274 (Proceedings of the Prehistoric Society 28, 1962).

54. Rodden, R. J. *et al.* Excavations at the Early Neolithic Site at Nea Nikomedeia, Greek Macedonia (1961 season). *Proc. Prehist. Soc.* **28**, 267–288 (1962).

55. Rodden, R. J. Recent discoveries from prehistoric Macedonia: an interim report. *Balk. Stud.* **5**, 109–124 (1964).

56. Marchi, N. *et al.* The genomic origins of the world’s first farmers. *Cell* **185**, 1842-1859.e18 (2022).

57. Maniatis, Y. Results of the radiocarbon samples from the Neolithic settlement of Stavroupoli Thessaloniki. in *Rescue excavations at the Neolithic site of Stavroupoli Thessaloniki* 847 (2002).

58. Kotsos, S. & Urem-Kotsou, D. Filling in the Neolithic landscape of central Macedonia, Greece. *Homage to Milutin Garasanin* 193–207 (2006).

59. Dushka, U.-K. & Kotsos, S. Settling Neolithic Central Macedonia, Northern Greece. in *Making Spaces into Places. The North Aegean, the Balkans and Western Anatolia in the Neolithic* 87–104 (BAR Publishing, 2020).

60. Lymperaki, M., Urem-Kotsou, D., Kotsos, S. & Kotsakis, K. Household Scales: What Cooking Pots Can Tell Us About Households in the Late Neolithic Stavroupoli (Northern Greece). *Open Archaeol.* **2**, 238–245 (2016).

61. Grammenos, D. B. & Kotsos, S. Rescue excavations at the Neolithic site of Stavroupoli Thessaloniki. *Thessaloniki Archaeol. Inst. North. Greece* **2**, (2002).

62. Urem-Kotsou, D. & Gioura, E. The pottery from new excavations. in *Rescue Excavations at Neolithic Settlement of Stavroupoli, Thessaloniki, Part II (1998-2003)* (eds. Grammenos, D. B. & Kotsos, S.) 219–303 (Archaeolological Institute of Northern Greece 6, 2004).

63. Alisøy, H. A. Consumption of ground stone tools at Stavroupoli. in *Rescue Excavations at Neolithic Settlement of Stavroupoli, Thessaloniki* (eds. Grammenos, D. B. & Kotsos, S.) 561–608 (Archaeolological Institute of Northern Greece 6, 2002).

64. Skourtopoulou, K. Chipped stone tools from the settlement of Stavroupoli. in *Rescue Excavations at Neolithic Settlement of Stavroupoli, Thessaloniki* (eds. Grammenos, D. B. & Kotsos, S.) 537–559 (Archaeolological Institute of Northern Greece 6, 2002).

65. Skourtopoulou, K. I lithotechnia tou apokrousmenou lithou ston oikismo tis Stavroupolis. in *Sostikes Anaskafes sto Neolithiko Oikismo Stavroupolis Thessalonikis, Part II* 361–476 (2004).

66. Hatzoudi. Preliminary study of bone artifacts. in *Rescue Excavations at Neolithic Settlement of Stavroupoli, Thessaloniki* (eds. Grammenos, D. B. & Kotsos, S.) 609–626 (Archaeolological Institute of Northern Greece 6, 2002).

67. Christidou, R. Note on the bone artefacts from Stavroupoli. in *Rescue Excavations at Neolithic Settlement of Stavroupoli, Thessaloniki* 477–488 (Archaeolological Institute of Northern Greece 6, 2002).

68. Efstratiou, N. *et al.* Excavations at the Neolithic settlement of Makri, Thrace, Greece (1988-1996). A preliminary report. in *Saguntum 31* 11–62 (1998).

69. Urem-Kotsou, D. & Efstratiou, N. I symvoli tis keramikis tipologias tis Makris sti meleti tisproistorikis ekseliksis sti Thraki. in *Archaeologiko Ergo sti Makedonia kai Thraki 7* 619–625 (1993).

70. Karkanas, P. & Efstratiou, N. Floor sequences in Neolithic Makri, Greece: micromorphology reveals cycles of renovation. *Antiquity* **83**, 955–957 (2009).

71. Hondroyianni-Metoki, A. Non domestic use of space in the Neolithic settlements. The example of Toumba Kremastis Koiladas. (Αριστοτέλειο Πανεπιστήμιο Θεσσαλονίκης (ΑΠΘ), 2009). doi:10.12681/eadd/19949.

72. Hondroyianni-Metoki, A. Egnatia odos, anaskafi stin proistoriki thesi ‘Toumba Kremastis Koiladas’-Nomou Kozanis. in *AEMTh 13* (ed. Adam-Veleni, P.) 399–414 (Ministry of Culture, 2001).

73. Pappa, M., Halstead, P., Kotsakis, K. & Urem-Kotsou, D. Evidence for large-scale feasting at Late Neolithic Makriyalos. in *Food, Cuisine and Society in Prehistoric Greece* 16–44 (2004).

74. Pappa, M. & Besios, M. The Neolithic settlement at Makriyalos, Northern Greece: preliminary report on the 1993–1995 excavations. *J. F. Archaeol.* **26**, 177–195 (1999).

75. Pappa, M. *DETh,Thermi, Makrygialos. The organization of communal and domestic space in the Neolithic settlements of Central Macedonia*. (Ziti, 2020).

76. Maniatis, Y. & Pappa, M. Radiocarbon Dating of the Neolithic Settlement at Makriyalos, Pieria, North Greece. *Radiocarbon* **62**, 467–483 (2020).

77. Maniatis, Y., Kotsakis, K. & Halstead, P. New AMS dates of the earliest Neolithic in Macedonia (Greece). in *AEMTh 25* (eds. Adam-Veleni, P. & Tsakalou, K.) 149–156 (Ministry of Culture, 2011).

78. Kotsakis, K. & Halstead, P. Anaskafi sta neolithika Paliambela Kolindrou. in *AEMTh 16* (ed. Adam-Veleni, P.) 407–415 (Ministry of Culture, 2004).

79. Halstead, P. & Kotsakis, K. Paliambela in Archaeological Reports for 2001-2002. 80 (2002).

80. Halstead, P. & Kotsakis, K. Paliambela in Archaeological Reports for 2002-2003. 46–66 (2003).

81. Triantaphyllou, S. Living with the Dead: a Re-consideration of Mortuary Practices in the Greek Neolithic. in *Escaping the Labyrinth: The Cretan Neolithic in Context* (eds. Isaakidou, V. & Tomkins, P.) 139–157 (Oxbow Monographs, 2008).

82. Ziota, C. The neolithic settlemment of “Kleitos I” and the new updates for prehistoric research. in *AEΑΜ* (ed. Karamitrou-Mentessidi, G.) 214–230 (2009).

83. Sampson, A. Late Neolithic remains at Tharrounia Euboea: a model for the seasonal use of settlements and caves. *BSA* **87**, 61–101 (1992).

84. Sampson, A. Proistorikon spileon Skotini para ta Tharrounia Euboeas. *AAA IX* 44 (1977).

85. Sampson, A. *Scoteini Tharrounion. To spilaio, o oikismos kai to nekrotapheio*. (Hellenic Ministry of Culture, 1993).

86. Theocharis, D. Nea Makri. Eine grossen neolithische Siedlung in der Nahe von Marathon. *Athen Mitteilungen* **71**, 1–29 (1956).

87. Coleman, J. *Keos I: Kephala. A Late Neolithic settlement and cemetery*. (Princeton University Press, 1977).

88. Sampson, A. I neolithiki katoikisi sto Giali tis Nisirou. (1988).

89. Sampson, A. I Neolithiki periodos sta Dodekanisa. (1987).

90. Stravopodi, E. An anthropological assessment of the human findings from the cave and the cemetery. in *Scoteini Tharrounion. To spileo, o oikismos kai to nekrotapheio* (Hellenic Ministry of Culture, 1993).

91. Nieszery, N. *Linearbandkeramische Gräberfelder in Bayern*. (verlag Marie Leidorf, 1995).

92. Dietrich, H. & Kociumaka, C. Jungsteinzeitliche Befunde aus Steinheim: Stadt und Landkreis Dillingen ad Donau, Schwaben. *Das Archäologische Jahr Bayern* 32–35 (2001).

93. Marchi, N. *et al.* The genomic origins of the world’s first farmers. *Cell* **185**, 1842-1859.e18 (2022).

94. Pechtl, J. Verorten in Raum, Zeit und Umwelt. Ein Forschungsprojekt zur ersten bäuerlichen Kultur in Bayerisch-Schwaben. *Denkmalpfl. Informationen* **161**, 14–17 (2015).

95. Brink-Kloke, H. Das linienbandkeramische Gräberfeld von Essenbach-Ammerbreite. *Germania* **68**, 427–481 (1990).

96. Scheu, A. *et al.* The genetic prehistory of domesticated cattle from their origin to the spread across Europe. *BMC Genet.* **16**, 54 (2015).

97. Unterländer, M. *et al.* Ancestry and demography and descendants of Iron Age nomads of the Eurasian Steppe. *Nat. Commun.* **8**, 14615 (2017).

98. Kircher, M., Sawyer, S. & Meyer, M. Double indexing overcomes inaccuracies in multiplex sequencing on the Illumina platform. *Nucleic Acids Res.* **40**, e3–e3 (2012).

99. Fu, Q. *et al.* A Revised Timescale for Human Evolution Based on Ancient Mitochondrial Genomes. *Curr. Biol.* **23**, 553–559 (2013).

100. Skoglund, P. *et al.* Separating endogenous ancient DNA from modern day contamination in a Siberian Neandertal. *Proc. Natl. Acad. Sci.* **111**, 2229–2234 (2014).
